# Spatial clustering of *Borrelia burgdorferi* sensu lato within populations of Allen's chipmunks and dusky-footed woodrats in northwestern California

**DOI:** 10.1371/journal.pone.0195586

**Published:** 2018-04-10

**Authors:** Gregory M. Hacker, Richard N. Brown, Natalia Fedorova, Yvette A. Girard, Mark Higley, Bernadette Clueit, Robert S. Lane

**Affiliations:** 1 Department of Natural Resources, Humboldt State University, Arcata, California, United States of America; 2 Department of Environmental Science, Policy and Management, University of California, Berkeley, California, United States of America; 3 Hoopa Tribal Forestry, Hoopa Valley Tribal Reservation, Hoopa, California, United States of America; Universita degli Studi di Parma, ITALY

## Abstract

The ecology of Lyme borreliosis is complex in northwestern California, with several potential reservoir hosts, tick vectors, and genospecies of *Borrelia burgdorferi* sensu lato. The primary objective of this study was to determine the fine-scale spatial distribution of different genospecies in four rodent species, the California ground squirrel (*Otospermophilus beecheyi*), northern flying squirrel (*Glaucomys sabrinus*), dusky-footed woodrat (*Neotoma fuscipes*), and Allen’s chipmunk (*Neotamias senex*). Rodents were live-trapped between June 2004 and May 2005 at the Hoopa Valley Tribal Reservation (HVTR) in Humboldt County, California. Ear-punch biopsies obtained from each rodent were tested by polymerase chain reaction (PCR) and sequencing analysis. The programs ArcGIS and SaTScan were used to examine the spatial distribution of genospecies. Multinomial log-linear models were used to model habitat and host-specific characteristics and their effect on the presence of each borrelial genospecies. The Akaike information criterion (AICc) was used to compare models and determine model fit. *Borrelia burgdorferi* sensu stricto was primarily associated with chipmunks and *B*. *bissettiae* largely with woodrats. The top model included the variables “host species”, “month”, and “elevation” (weight = 0.84). Spatial clustering of *B*. *bissettiae* was detected in the northwestern section of the HVTR, whereas *B*. *burgdorferi* sensu stricto was clustered in the southeastern section. We conclude that the spatial distribution of these borreliae are driven at least in part by host species, time-of-year, and elevation.

## Introduction

Lyme borreliosis is currently recognized as the most commonly reported vector-borne disease of humans in the United States [1]. It is caused by a group of more than 20 related genospecies of bacteria referred to as *Borrelia burgdorferi* sensu lato (s. l.) that are transmitted to vertebrate hosts via the bite of *Ixodes* spp. ticks [2–4]. In North America, there is considerable diversity among genospecies within the *B*. *burgdorferi* s. l. complex, but only a small subset is known to cause Lyme borreliosis in humans [5–11]. Recent studies have characterized previously ungrouped genospecies belonging to the *B*. *burgdorferi* s. l. group, such as *B*. *bissettiae* [12–13], *B*. *californiensis* [14], *B*. *carolinensis* [15], *B*. *americana* [16], *B*. *kurtenbachii* [4, 17], and *B*. *lanei* [18]. The only genospecies known to cause Lyme borreliosis in humans in North America was *B*. *burgdorferi* sensu stricto (s. s.) until recently [10, 19]. That changed in 2011, when *B*. *bissettiae* DNA was detected in human sera and subsequently in cardiac tissue indicating its importance as a potential disease-causing agent in California and the southeastern United States [20–21]. More recently, *B*. *americana* and *B*. *andersonii* were incriminated as human pathogens in the southeastern United States [5], and *B*. *mayonii* was found to cause clinical disease in the upper Midwestern United States [11]. According to Clark et al. [6], *B*. *americana*-like strains have also been recovered from patients in the northeastern, northwestern, southwestern, and southeastern United States. The latter findings await confirmation, however.

In California, the ecology of Lyme borreliosis spirochetes is more complex, and therefore less understood, than its ecology in the northeastern United States [3, 22–24] because of multiple potential reservoir hosts, tick vectors, and genospecies of *B*. *burgdorferi* s. l. [7, 13, 19, 25–31]. In the far-western United States, *B*. *burgdorferi* s. s. is transmitted to humans by the western black-legged tick (*Ixodes pacificus*) [32–33]. Other enzootic cycles involving ticks and spirochetes other than *I*. *pacificus* and *B*. *burgdorferi* s. s. have been described [24–26]. For example, *Ixodes spinipalpis* was initially thought to contribute to the enzootic maintenance of *B*. *burgdorferi* s. s., but that non-human-biting tick is now known to be more important in the enzootic maintenance of *B*. *bissettiae* [24–25, 27, 34–35].

Several species of rodents are important reservoirs of *B*. *burgdorferi* s. l. in California including the California kangaroo rat (*Dipodomys californicus*), the western gray squirrel (*Sciurus griseus*), and the dusky-footed woodrat. The dusky-footed woodrat is the primary reservoir host in an enzootic cycle involving *B*. *bissettiae* and *I*. *spinipalpis* [25, 27–28, 31, 34–35]. Similarly, there is a high prevalence (30% to 80%) of infection of *B*. *burgdorferi* s. s. in western gray squirrels in oak-woodland habitats in northwestern California [30, 36–37], which suggests that other sciurid species occupying similar niches likewise may support cycles of *B*. *burgdorferi* s. s. in ecologically diverse California. One such group, the chipmunks (*Neotamias* spp.) are likely candidates–they are active diurnally and occupy a range of habitats from coastal rain forests to arid sagebrush steppe, many of which overlap the biotopes of *B burgdorferi* s. l. and *I*. *pacificus* [38–39]. Both redwood (*Neotamias ochrogenys*) and Siskiyou (*Neotamias siskiyou*) chipmunks reportedly host *B*. *burgdorferi* s. l. in California [40], hinting at their possible importance in the maintenance of such spirochetes. For rodents that inhabit forested environs, any activity that fragments the landscape and alters the composition of a forest (e.g., logging, fire, or disease) also may affect the distribution and density of rodents, their associated ticks, and pathogens [38–39, 41–51].

Forestry practices in California have created a mosaic-like landscape that differs dramatically from that prior to European settlement [41]. Such a patchwork of habitats doubtless has influenced the contemporary ecology of *B*. *burgdorferi* s. l [48–49]. Landscapes managed for timber generally consist of forest units classified by the number of years since the most recent harvest. Structural stage is defined as the stage of a forest unit that is related to the size and orientation (vertical and horizontal) of trees and tree limbs [42]. Forest management practices often result in complex forest structure depending on the intended goals of the management, ranging from clear-cut units and units with simple structure to older forest units with complex structure. The response of many rodent communities to forest management practices depends on a variety of factors including plant species composition and forest structure [43–46], the amount of downed woody debris [47], and stand age since last harvest. These factors also may impact the presence or absence of pathogens in rodent communities. In New York State, for example, a greater prevalence of *B*. *burgdorferi* s. l. in *Ixodes scapularis* nymphs was associated with smaller forest units, which may have a direct impact on the prevalence of *B*. *burgdorferi* s. l. in rodents [48].

The focal nature of many vector-borne diseases is typically due to the interactive effects of various biotic and abiotic processes resulting in temporal and spatial clustering in hosts and vectors. In California, the density of questing *I*. *pacificus* nymphs irrespective of their infection status with *B*. *burgdorferi* s. l., was found to vary both temporally and spatially [49–50]. This indicates that risk of human exposure to this pathogen is driven in part by location and time-of-year. By extending these findings to rodents, we ask whether *B*. *burgdorferi* s. l. similarly may be clustered within rodent populations. Hence, to further evaluate the role of rodents in the ecology of *B*. *burgdorferi* s. l. in forested areas, we evaluated environmental factors that may affect the distribution of Lyme borreliosis spirochetes within known or potential rodent reservoirs in northwestern California. To that end, we sought to compare the prevalence of *B*. *burgdorferi* s. l. among rodent species at the Hoopa Valley Tribal Reservation (HVTR) in northwestern California to determine whether rodents infected with *B*. *burgdorferi* s. l. are geographically clustered, and to ascertain if host specific and/or forest structure attributes influence the prevalence and spatial distribution of *B*. *burgdorferi* s. l.

## Materials and methods

### Study site

The HVTR (Latitude = 41.049141, Longitude = -123.677064) encompasses approximately 360 km^2^ in northern Humboldt County. The climate is characterized by cool, wet winters and warm, dry summers. The reservation is bisected by the Trinity River that runs south to north and flows into the Klamath River to the north. Elevation ranges from 100 m above sea level in the Trinity River valley to more than 1,500 m above sea level along its eastern border. The principal habitat type is montane hardwood-conifer forest with dominant tree species including Douglas fir (*Pseudotsuga menziesii)*, tanoak (*Notholithocarpus densiflorus*), incense cedar (*Calocedrus decurrens*), Pacific madrone (*Arbutus menziesii*), California black oak (*Quercus kelloggii*), and Oregon white oak (*Q*. *garryana*). Evergreen huckleberry (*Vaccinium ovatum*) and salal (*Gaultheria shallon*) are the dominant understory shrubs [41]. Recent forestry practices guided by the tribe’s Forest Management Plan include the retention of snags (dead trees) and large trees, and “thin and release” practices in which a subset of trees are thinned from a forest unit to decrease competition and accelerate tree growth. The result of these forestry practices is a mixture of old growth units (more than 80 years post-harvest) and second growth units (up to 80 years post-harvest) having increased structure compared to typical clear-cuts (Table 1). Access to the HVTR was granted by Hoopa Tribal Forestry and the Hoopa Tribal Council.

**Table 1:** Forest unit categories and descriptions used to describe individual forest units.

Unit Category	Code	Description
Old Forest	OF	Common stage for non-harvested forest units and some older (> 80 years post-harvest) harvested forest units that have large numbers of residual large trees.
Young Forest Multi-storied	YFM	Patches of tree mortality cause a young forest unit to differentiate into multiple strata with varying heights (i.e., understory, mid-story, and canopy). Tree mortality opens up the canopy allowing for increased understory growth and accelerated growth of over-story trees near openings. Common plant species are similar to OF.
Stem Exclusion	SX	Young regenerating forest in which trees initially grow quickly, but growth slows as competition for resources increases. Tree crowns merge reducing the number of shade intolerant shrubs. Because of management practices, most SX units used in this study consisted primarily of young Douglas fir.
Understory Re-initiation (Stem Exclusion)	UR(SX)	Older multi-storied forest that usually results from cuts with considerable large tree retention, uneven age management, or low to moderate intensity fire. This stage has a dense understory canopy and a few shade intolerant shrubs. Vegetation composition is similar to YFM and other forest units.
Brushy Pole and Pole Unit	BP	A general term usually indicating a young regenerating forest resulting from fire or clear cut. This category is the result of joining two similar forest unit types and comprises structural stages that include brushy pole units where new seedlings establish into a dense layer of brush and saplings with no over-story, and young pole units where new seedlings develop into a dense layer of saplings. Canopy species, if present, are Douglas fir, incense cedar, or tan-oak.
Non-forested	NF	Naturally occurring areas of prairie, meadow, or wetland.

### Small mammal sampling

Rodents were trapped during 24 sampling occasions from June 2004 to May 2005. Sampling occasions lasted from 1–5 days, depending on weather, and all sites were sampled only once. Traps (Tomahawk brand, Tomahawk Live Trap Company, Tomahawk, WI; and Sherman brand, H. B. Sherman Traps, Tallahassee, FL) were placed at locations that maximized the probability of capturing the following rodent species: the dusky-footed woodrat, Allen’s chipmunk, and the California ground squirrel. Typically, one Sherman and one Tomahawk trap was set per sampling site near rodent burrows. Traps were checked at dawn for overnight sampling sessions and were checked every four hours during daytime sampling. Variation in the size of sampling sites prevented the use of equal numbers of traps in all locations. All animal handling methods were approved by the Humboldt State University Institutional Animal Care and Use Committee. Captured animals were anesthetized with a subcutaneous injection of ketamine hydrochloride (40 mg/kg). Two 2-mm ear punch biopsies (EPBs) were collected from each captured animal (one per pinna) and stored in 95% ethanol until they could be tested for presence of borreliae. Species, sex, age, morphometric measurements, and location of capture (GPS coordinates) were collected from each rodent. Monel metal-fingerling tags (National Band and Tag Company, Newport, KY) were used to uniquely mark both ears of all animals captured. Animals were released at their sites of capture following recovery from anesthesia. Recaptured animals were noted and immediately released at their point of capture. Data on tick abundance on hosts and infection status were not gathered during this study.

### Genetic analysis

Molecular analyses were conducted in the Lane Laboratory at the University of California, Berkeley. Genomic DNA was extracted from ear tissues using the DNeasy Blood and Tissue Kit (Qiagen, Valencia, CA) according to the manufacturer’s protocol with overnight digestion in ATL buffer with proteinase K at 56°C. Amplification of the 5S-23S rRNA intergenic spacer region of *Borrelia burgdorferi* s. l. was performed as described previously [52]. Multiple negative (deionized water) and positive controls (*B*. *burgdorferi* isolate CA4) were included with each PCR run. Products from positive samples were purified using the QIAquick PCR purification kit (Qiagen, Valencia, CA). Sequencing was performed at the University of California, Berkeley DNA Sequencing Facility using previously described nested PCR primers [52]. Sequences were assembled and manually edited using Sequencher 4.5 (Gene Codes Corp., Ann Arbor, MI). A 158 base-pair PCR product was used for phylogenetic analysis by the neighbor-joining method implemented in PAUP* 4.0 (uncorrected *p* distances) [53] and sequences were determined to belong to *Borrelia* genospecies based on similarity to sequences available in GenBank (National Center for Biotechnology Information, 2008). Amplicons that could not be classified to genospecies due to mixed sequences were eliminated from further phylogenetic analysis and categorized as unclassified *B*. *burgdorferi* s. l.

### Spatial analysis

The programs ArcGIS (version 10.0, Environmental Systems Research Institute, Inc., Redlands, CA) and SaTScan (version 9.0, www.satscan.org, accessed 3 January 2012) were used to assess the spatial relationships between hosts and associated borreliae [54]. SaTScan uses a spatial scan statistic to identify the most likely clusters of rodents infected with *B*. *burgdorferi* s. s. or *B*. *bissettiae*. An elliptical scanning window was used to include clusters with shapes other than circles to allow for differences in geography (i.e., ellipses that run along riparian corridors or mountain ridges). The Bernoulli model was used to assess clustering of each borrelial genospecies within rodent hosts. To evaluate both small and large clusters, the maximum number of capture locations (i.e., population) within a cluster was set from 35% to 50% of the total samples and an alpha of 0.05 was set as the criterion for significance in all tests. By setting the maximum population size to 35% of the total, we restricted the size of clusters so they do not cross the Trinity River. ArcGIS was used to map statistically significant clusters and individual locations of borrelial infections in rodents across the HVTR.

### Habitat and host specific analyses

An information-theoretic approach using a priori-selected multinomial log-linear models (program R, version 3.1.1) and predictor variables was used to evaluate the effects of landscape and host-specific variables on the presence or absence of *B*. *burgdorferi* s. s. and *B*. *bissettiae* in rodent hosts. Variables used as predictors of infection status included the elevation of capture site, month of capture, forest unit of capture (i.e., structural stage), dominant overstory vegetation, dominant understory vegetation, area of the forest unit surrounding the capture location (m^2^), host species, host sex, host age (juvenile, subadult, or adult), and host weight. Rodent species that yielded no PCR-positive results were subsequently removed from further analysis. The variable “forest unit” consisted of seven categories corresponding to differences in structural stage (Table 1).

Akaike's Information Criterion corrected for small sample sizes (AICc) was used to select the best fitting model with the greatest predictive power. From the top ranked model, coefficients of variation were analyzed along with standard errors to determine which variables positively or negatively influenced the prevalence of each genospecies. The predicted probabilities of infection class (i.e., not infected, infected with *B*. *burgdorferi* s. s., or *B*. *bissettiae*) were calculated from the exponentiated coefficients (i.e., relative risk ratios) of individual variables obtained from the output of the top model. The predicted probabilities produced by the top model were used to determine the association between combinations of predictor variables and their effect on the probability of infection of an individual rodent.

## Results

**Fig 1:**
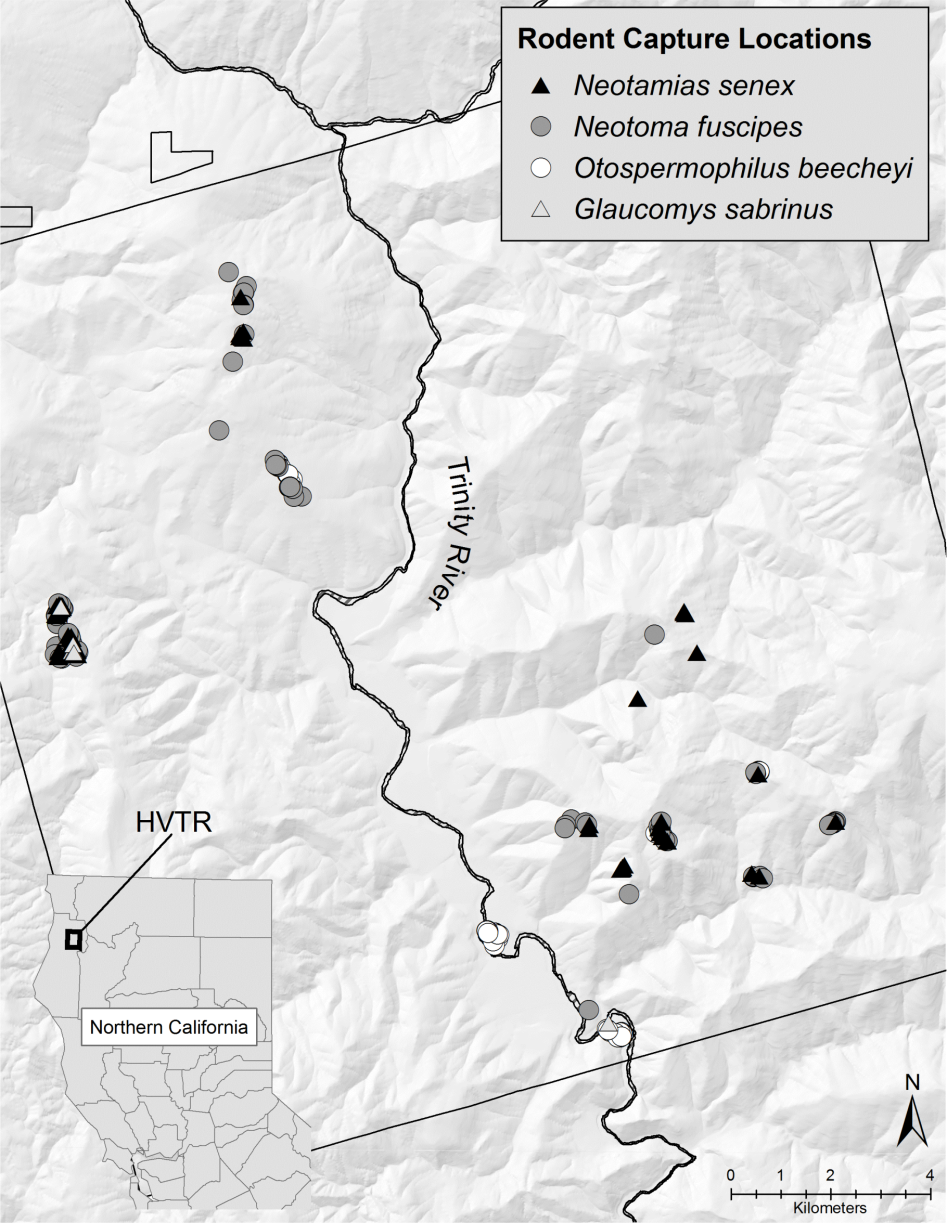
Locations of California ground squirrels (*O*. *beecheyi*), northern flying squirrels (*G*. *sabrinus*), dusky-footed woodrats (*N*. *fuscipes*), and Allen’s chipmunks (*N*. *senex*) captured and tested for the presence of *B*. *burgdorferi* s. l. within the HVTR from June 2004 to May 2005. Each location represents one to many individuals because of overlapping capture locations.

A total of 284 rodents were trapped and tested including 74 California ground squirrels, seven northern flying squirrels, 114 dusky-footed woodrats, and 84 Allen’s chipmunks (hereinafter referred to as woodrats and chipmunks) (Fig 1). All California ground squirrels and northern flying squirrels were PCR negative for *B*. *burgdorferi* s. l. and excluded from subsequent analyses (Table 1). Overall, the prevalence of woodrats and chipmunks PCR-positive for *B*. *burgdorferi* s.l. was similar (Table 2). However, woodrats had a higher prevalence of *B*. *bissettiae*, whereas chipmunks had a higher prevalence of *B*. *burgdorferi* s. s. (Table 2).

**Table 2:** Numbers of Allen’s chipmunks (*N*. *senex*), dusky-footed woodrats (*N*. *fuscipes*), California ground squirrels (*O*. *beecheyi*), and northern flying squirrels (*G*. *sabrinus*) trapped at the HVTR from June 2004 through May 2005 and infection prevalence with Bbss (*B*. *burgdorferi* s. s.), Bbis (*B*. *bissettiae*), or unclassified Bbsl (*B*. *burgdorferi* s. l.).

Species	Number Trapped	Number (%) Bbss	Number (%) Bbis	Number (%) Unclass. Bbsl
Allen’s Chipmunk	84	14 (16.7%)	7 (8.3%)	0
Dusky-footed Woodrat	114	4 (3.5%)	23 (20.2%)	6 (5.3%)
California Ground Squirrel	74	0 (0%)	0 (0%)	0 (0%)
Northern Flying Squirrel	7	0 (0%)	0 (0%)	0 (0%)

### Spatial analysis

**Fig 2:**
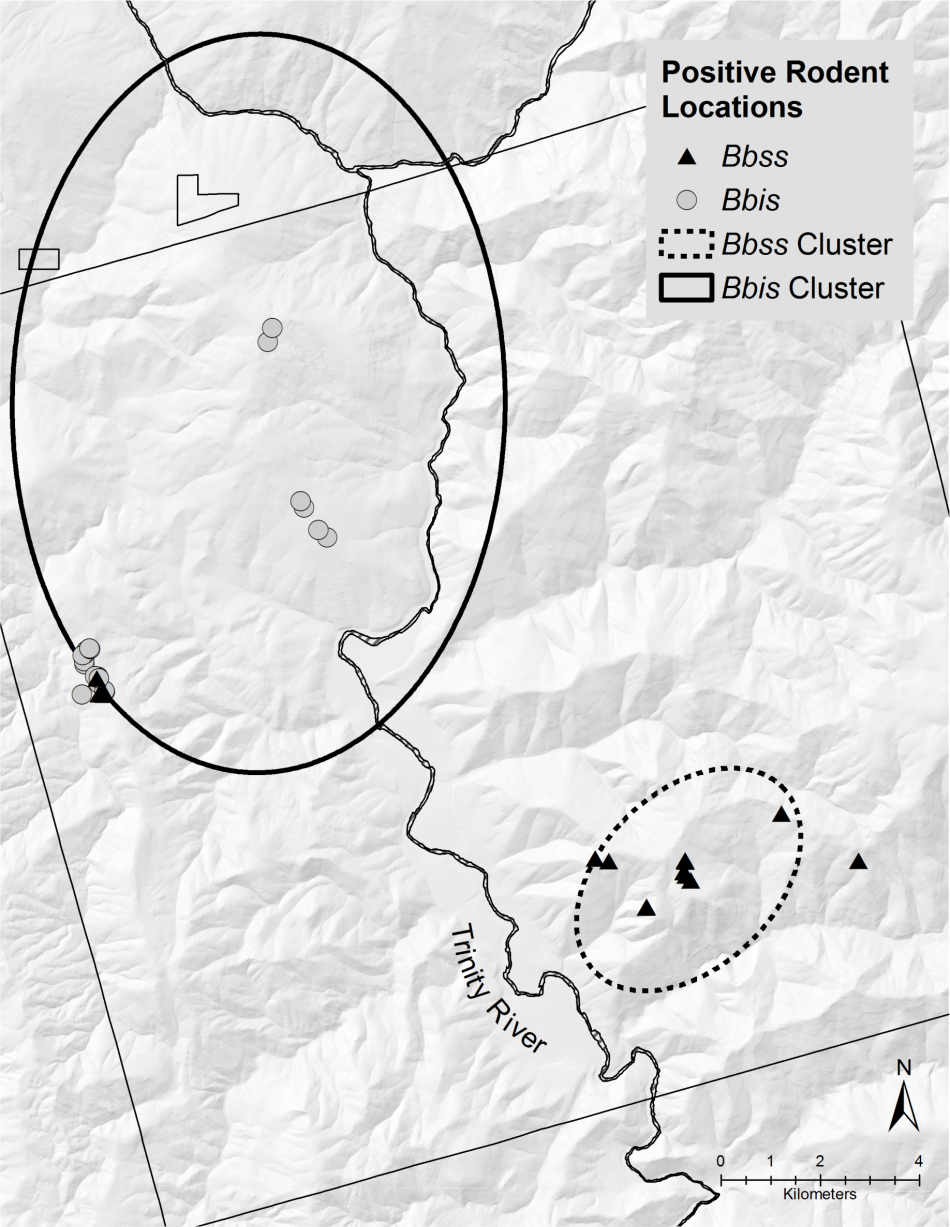
Locations of Allen’s chipmunks (*N*. *senex*) and dusky-footed woodrats (*N*. *fuscipes*) infected with *B*. *burgdorferi* s. s. (Bbss) or *B*. *bissettiae* (Bbis) and associated significant clusters within the HVTR.

A total of 55 chipmunks and 74 woodrats were trapped in the northwestern section of the HVTR. A greater proportion of woodrats (n = 23, 31.1%) were positive for *B*. *bissettiae* as compared to chipmunks (n = 7, 12.7%). A cluster of 22 woodrats and 7 chipmunks PCR-positive for *B*. *bissettiae* (total population, 104) was detected in the northwestern section (*RR* = 9.01, *P*<0.001) (Fig 2). Five (9.1%) chipmunks also were positive for *B*. *burgdorferi* s. s in the northwestern section. All six unclassified *B*. *burgdorferi* s. l. samples were collected from woodrats in the northwestern section.

A total of 29 chipmunks and 45 woodrats were trapped in the southeastern section of the HVTR. Nine of 29 (31.0%) chipmunks trapped in the southeastern section were positive for *B*. *burgdorferi* s. s. and none were positive for *B*. *bissettiae*. Additionally, four (8.9%) of 45 woodrats were positive for *B*. *burgdorferi* s. s. No woodrats trapped in the southeastern section of the HVTR were positive for *B*. *bissettiae*. A cluster of 9 chipmunks and 3 woodrats PCR-positive for *B*. *burgdorferi* s. s. (total population, 42) was detected in the southeastern section including (*RR* = 8.1, *P* = 0.004) (Fig 2).

### Habitat and host specific analyses

The top ranked model accounted for 84% of the cumulative weight of a model set that consisted of 32 models and included the variables “species”, “month”, and “elevation” (Tables 3 and 4, S3 Table). The fourth ranked model had the largest model likelihood (i.e. greatest fit) versus the fully parameterized model and included the same variables as the top model, with the addition of the “forest unit” variable.

**Table 3:** The top six of 32 multinomial log-linear models analyzed. Models were ranked via a corrected Akaike’s Information Criterion (AICc) value. The K* column indicates the number of parameters for each model.

Model	K*	LogL	AICc	Δ AIC	Model Weight	Cum. Weight
Species + Month + Elevation	18	-106.66	253.14	0.00	0.84	0.84
Species + Elevation	6	-122.34	257.11	3.97	0.12	0.96
Forest Unit + Species + Elevation	16	-112.21	259.42	6.28	0.04	0.99
Forest Unit + Species + Month + Elevation	28	-99.41	264.42	11.28	0.00	0.99
Forest Unit + Elevation	14	-117.46	265.21	12.07	0.00	0.99
Forest Unit + Area of Unit + Elevation	16	-115.85	266.71	13.57	0.00	0.99

**Table 4:** Coefficients, associated standard errors, and relative risk ratios from the top multinomial log-linear model as ranked by AICc. The abbreviations Bbss and Bbis stand for *Borrelia burgdorferi* sensu stricto and *Borrelia bissettiae*, respectively. The term “Chipmunk” refers to the Allen’s Chipmunk (*N*. *senex*).

Response	Intercept	Chipmunk	June	July	August	February	March	May	Elevation
Bbss	-11.99	2.52	0.79	0.58	-0.13	-9.94	-30.78	-1.06	0.01
Std. error	0.69	0.60	1.18	1.02	1.25	0.00	0.00	1.41	0.001
Bbis	1.22	0.49	0.00	0.57	2.57	0.00	0.81	0.00	0.002
Std. error	0.21	0.49	0.00	0.25	0.00	0.00	0.69	0.00	0.34
Relative Risk Ratios									
Bbss	0.00	12.45	2.20	1.79	0.87	0.00	0.00	0.35	1.01
Bbis	2.60	0.42	0.00	0.99	0.00	0.00	1.69	0.00	0.99

Analyses of relative risk ratios and predicted probabilities from the top ranked model demonstrate that, compared to chipmunks, woodrats were more often associated with infection with *B*. *bissettiae* than not infected (RR = 2.6, Table 4). Compared to woodrats, chipmunks were more often associated with *B*. *burgdorferi* s. s. infection than not infected (RR = 12.5, Table 4).

**Fig 3:**
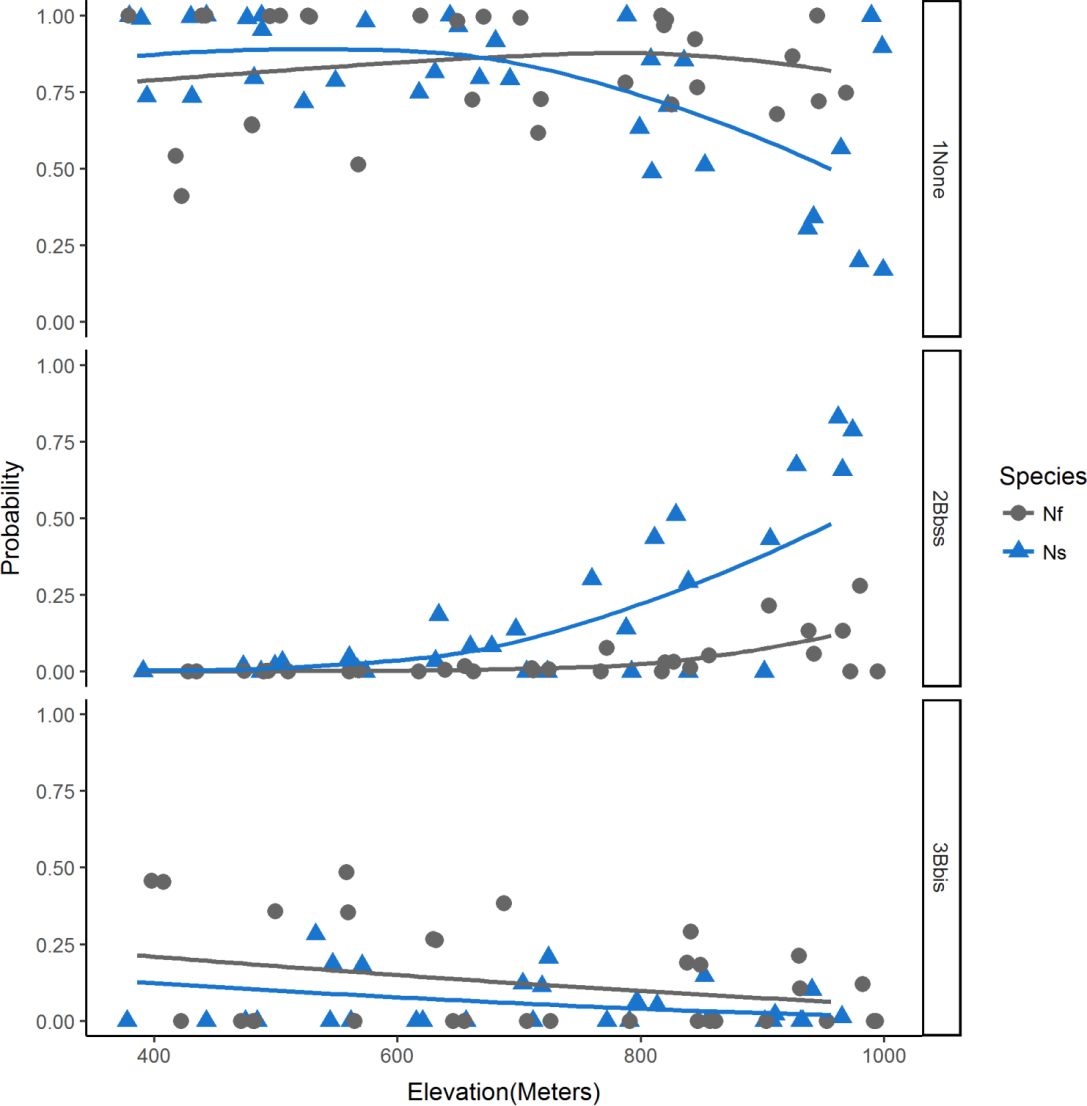
The predicted probabilities of no infection (1None), infection with *B*. *burgdorferi* s. s. (2Bbss), or *B*. *bissettiae* (3Bbis) from (a) 350 m to 1000 m above sea level at the HVTR. Blue triangles represent probabilities associated with Allen’s chipmunks (Ns, *Neotamias senex*) and gray circles represent probabilities associated with dusky-footed woodrats (Nf, *Neotoma fuscipes*). LOESS curves (solid blue and gray lines) were used to display the relationship between probability of infection, elevation, and host species.

Elevation and month of capture contributed significantly to the top model (Tables 3 and 4). Woodrats were trapped at elevations ranging from 175 m to 964 m (average = 723 m). Chipmunks were trapped at elevations ranging from 447 m to 961 m (average = 704 m). Additionally, the average elevation of rodents trapped in the northwestern section of the HVTR was 658 m while the average elevation of rodents trapped in the southeastern section of the HVTR was 818 m. The average elevation at which *B*. *burgdorferi* s. s.-positive rodents were trapped throughout the HVTR was 827 m (range = 638–961 m), whereas *B*. *bissettiae*-positive rodents (all in the northwestern section of HVTR) were trapped at an average elevation of 643 m (range = 514–923 m). *Borrelia burgdorferi* s. s.-positive rodents in the southeastern section were trapped at an average elevation of 891 m. Our top model revealed that, as elevation increased the relative risk of observing a rodent infected with *B*. *burgdorferi* s. s. also increased (RR = 1.01, Table 4). This pattern was amplified when examining the predicted probabilities of infection with *B*. *burgdorferi* s. s., given a chipmunk host, and increasing elevation (Fig 3). The relative risk of a rodent infection with *B*. *bissettiae* decreased with increasing elevation (RR = .99) (Table 4). A negative relationship was observed between the predicted probabilities of infection with *B*. *bissettiae* for woodrats and increasing elevation (Fig 3).

**Fig 4:**
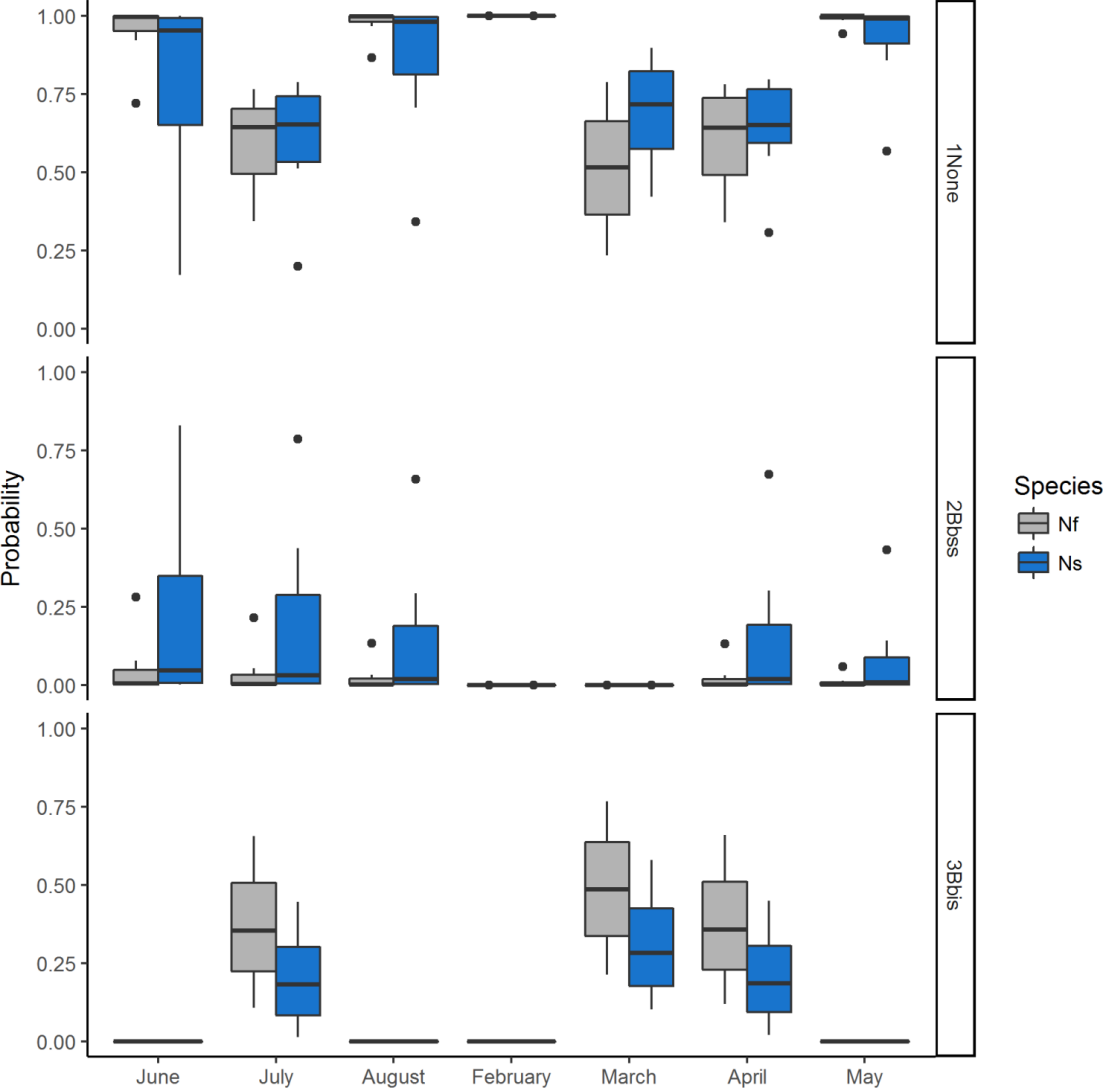
The predicted probabilities of no infection (1None), infection with *B*. *burgdorferi* s. s. (2Bbss), or *B*. *bissettiae* (3Bbis) by month at the HVTR. Blue bars represent probabilities associated with Allen’s chipmunks (Ns, *Neotamias senex*) and gray bars represent probabilities associated with dusky-footed woodrats (Nf, *Neotoma fuscipes*).

The relative risk of infection and associated predicted probabilities of infection with *B*. *burgdorferi* s. s. were greatest in the summer months (June–August) with another peak in April (Table 4, Fig 4). In contrast, the relative risk and associated predicted probabilities of infection with *B*. *bissettiae* were greatest in the spring months with another peak in July (Table 4, Fig 4). The greatest probability of infection with *B*. *burgdorferi* s. s. was associated with a chipmunk host, summer months, and higher elevations, whereas the greatest probability of infection with *B*. *bissettiae* was associated with a woodrat host, spring months, and lower elevations.

## Discussion

Here, we report for the first time a high prevalence of infection with *B*. *burgdorferi* s. s. and the highest reported prevalence of *B*. *bissettiae* in Allen’s chipmunks in the western United States. Our findings demonstrate that at broad and fine scales, Allen’s chipmunks not only host these pathogens, but are likely to be integral in maintaining and distributing them (Tables 2 and 4). The prevalence of *B*. *bissettiae* observed in dusky-footed woodrats mirrors that reported previously for this rodent from chaparral and oak woodland habitats in northern California, and reconfirms the important role of woodrats in the enzootic maintenance of this spirochete [25,27–29, 31, 34]. Two of the six named genospecies, i.e., *B*. *burgdorferi* s. s., *B*. *bissettiae*, *B*. *americana*, *B*. *californiensis*, *B*. *carolinensis*, and *B*. *lanei* (formerly *B*. genomospecies 2) known to circulate in California were detected in this study. This compares favorably with a previous report that found seven genospecies of *Borrelia burgdorferi* s. l. including one novel borrelial spirochete in Alameda County, California [7]. The six unclassified *B*. *burgdorferi* s. l. samples from woodrats had amplicons with nucleotide mixtures at one or more positions. This suggests that these woodrats were possibly infected with a mixture (i.e., co-infection) of *B*. *burgdorferi* s. s. and *B*. *bissettiae*, or mixtures of other borrelial genospecies known or heretofore unknown to circulate in California. However, further characterization of these genospecies was not possible for these six samples. The use of cloning techniques may have aided in the identification of genospecies in these samples, but were not used during this study.

### Spatial analysis

This study revealed significant spatial segregation of *B*. *burgdorferi* s. l. within two rodent populations at the HVTR, which likely is due to the abundance and distribution of these rodents and their associated ticks across the landscape. In the northwestern cluster, the presence of *B*. *bissettiae* in both woodrats and chipmunks may demonstrate an ecological overlap between these species (Figs 1 and 2). The increased prevalence of *B*. *bissettiae* in woodrats versus chipmunks reconfirms that woodrats serve as the primary reservoir host for *B*. *bissettiae* [7, 25, 27–29, 31, 34, 55]. We conclude that biotic and abiotic factors affecting the distribution of woodrats likely determine the distribution and clustering of *B*. *bissettiae*.

A high prevalence of *B*. *burgdorferi* s. s. infection (31.0%) within the chipmunk population in the southeastern cluster contrasts with the relatively low prevalence (9.1%) in chipmunks throughout the rest of the HVTR. Additionally, the only woodrats PCR-positive for *B*. *burgdorferi* s. s. throughout the HVTR were from the southeastern cluster. This is an important finding because *B*. *burgdorferi* s. s. seldom has been observed in woodrats [27, 31, 56] as compared with *B*. *bissettiae* [27, 31, 34]. A lack of *B*. *bissettiae*-positive rodents in the southeastern section of the HVTR could be the result of limited sampling. This seems unlikely, however, and we posit instead that environmental differences between the two clusters are more likely to account for the absence of *B*. *bissettiae*. Although beyond the scope of this study, the use of population genetics approaches may provide deeper insights into the distribution and possible evolution of borreliae within rodents and ticks at fine spatial scales.

A concurrent study at the HVTR identified a relatively high seroprevalence of *Anaplasma phagocytophilum*, the causative agent of human granulocytic anaplasmosis, in dusky-footed woodrats, Allen’s chipmunks, western gray squirrels (*Sciurus griseus*) and Douglas squirrels (*Tamiasciurus douglasii*) [57]. Further, areas west of the Trinity River were more likely to have seropositive animals than east of the river [57]. The cluster of *B*. *bissettiae*-positive rodents observed in the current study overlaps the cluster of *A*. *phagocytophilum* seropositive samples. Hence, there may be similar environmental processes at play that influence the distribution of *B*. *bissettiiae* and *A*. *phagocytophilum*. We therefore examined some local environmental and host-specific variables to determine their possible effects on the prevalence and spatial clustering of *B*. *burgdorferi* s. l.

### Habitat and host specific analyses

The coefficients and predicted probabilities from the top ranked model demonstrate a clear association between woodrats and *B*. *bissettiae*, and chipmunks and *B*. *burgdorferi* s. s. (Tables 3 and 4). The low prevalence of *B*. *bissettiae* in chipmunks and *B*. *burgdorferi* s. s. in woodrats underscores the complexity of this system in northern California, and indicates that these transmission cycles are interdependent because of shared hosts and vector ticks. In an earlier study at the HVTR, Allen’s chipmunks were more often parasitized by *I*. *pacificus* than by any other tick species [46], which may partially explain the high prevalence of *B*. *burgdorferi* s. s. we detected in that rodent. Prior research also has demonstrated low prevalences of *B*. *bissettiae* in *I*. *pacificus* and *B*. *burgdorferi* s. s. in *I*. *spinipalpis* [27], likely accounting for the occasional presence of *B*. *burgdorferi* s. s. in woodrats and *B*. *bissettiae* in chipmunks observed in our study. The high prevalence of *B*. *burgdorferi* s. s. in Allen’s chipmunks and *B*. *burgdorferi* s. l. in other chipmunk species suggests that chipmunks may serve as primary reservoir hosts of such spirochetes [40, 58–59].

The spatial clustering of *B*. *burgdorferi* s. l. at the HVTR appears to be driven by the abundance and distribution of woodrats and chipmunks. However, similar proportions of both rodents were trapped in the northwestern and southeastern sections of the HVTR. If clustering of either genospecies is determined solely by the abundance or density of hosts, then similar proportions of infected individuals would be expected in both areas. However, *B*. *burgdorferi* s. s.–infected woodrats were only trapped in the southeastern section of the HVTR, suggesting a link between the increased prevalence in local chipmunks and its occasional presence in woodrats. Conversely, no woodrats infected with *B*. *burgdorferi* s. s. were trapped in the northwestern section of the HVTR suggesting that woodrats are not a primary reservoir for *B*. *burgdorferi* s. s. there. The observed spatial clustering cannot be explained adequately by differences in the relative abundance of hosts and, therefore it must be driven, at least partly, by other factors.

The inclusion of “month” in the top model indicates a significant temporal association between the presence of *B*. *burgdorferi* s. l. and rodents. This may be the result of temporal activity patterns of rodent hosts (Fig 4) and their associated tick vectors. Chipmunk activity generally increases in the late spring and summer months, whereas woodrats are active throughout the year [60–61]. However, it would be difficult to discern the difference between temporal activity patterns of hosts, and a pattern where hosts acquire infections of different genospecies at different times of the year. The temporal activity patterns of *I*. *pacificus* and *I*. *spinipalpis* also are important in the transmission of *B*. *burgdorferi* s. l. to rodent hosts [35, 49–50, 62–67]. *Ixodes pacificus* nymphs feed primarily on lizards and rodents and are active from late winter to early summer [30, 32–33, 50, 68], matching the prevalence of *B*. *burgdorferi* s. s. observed in rodents in this study (Fig 4). Activity of *I*. *spinipalpis* nymphs and larvae on rodent hosts has been shown to peak in the fall to spring months [28, 35], coinciding with the peak prevalence of *B*. *bissettiae* observed in rodents in this study (Fig 4). The diel activity patterns of woodrats (nocturnal) and chipmunks (diurnal) may more closely correspond to differing diel patterns of questing *I*. *spinipalpis* and *I*. *pacificus* and may determine the species of tick most likely to be attached to a given host [38, 60–61, 65].

The elevation at which a rodent was captured also was a significant predictor of the presence of *B*. *burgdorferi* s. l. in rodents. Similar numbers of woodrats and chipmunks were captured in most sampled areas at the HVTR. Therefore, the increased probability of infection with *B*. *burgdorferi* s. s. at higher elevations and *B*. *bissettiae* at lower elevations (Table 4, Fig 3) cannot be adequately explained by the distribution of hosts, dominance of a host at a site, or a greater number of samples at a given elevation.

Forest units west of the Trinity River generally are lower in elevation and more humid than eastern units that tend to be higher in elevation and drier (Fig 1). Both *B*. *burgdorferi* s. s. and *B*. *bissettiae*-infected rodents were found in areas west of the Trinity River, but only *B*. *bissettiae* was found to significantly cluster in rodents west of the river at a lower average elevation than the eastern side of the HVTR. Similarly, only *B*. *burgdorferi* s. s.-positive rodents were found to cluster on the eastern portion of the HVTR at a higher average elevation than those sampled throughout the HVTR. This pattern may be multi-factorial including differences in local climate [66–67], habitat characteristics [27–28, 69], and their effect on the distribution and abundance of reservoir hosts and primary vectors. In Alameda County, California, *B*. *burgdorferi* s. s. was more prevalent in ticks from warmer and drier areas, whereas *B*. *bissettiae*-infected *I*. *pacificus* ticks were more prevalent in the cooler, more humid environments influenced by the maritime conditions in San Francisco Bay [7]. A significant positive association exists between elevation and *I*. *pacificus* abundance and *B*. *burgdorferi* s. l. prevalence in California, likely due to the effect of temperature and humidity on tick abundance at different elevations [66–67]. This association may help explain the presence and clustering of *B*. *burgdorferi* s. s. in woodrats and chipmunks observed in the southeastern cluster.

Although “forest unit” was not included in the top model, it was included in four of the top six models suggesting that it had some predictive value. Furthermore, the fourth most competitive model that contained the “forest unit” variable had the greatest model likelihood, indicating that it best fit the data, but was penalized by AICc because of small sample sizes and additional parameters resulting from the classification of forest unit structural stages. Overall, the abundance of woodrats and chipmunks caught in each forest unit was expected because of each host’s known habitat preferences [38, 43, 46, 47, 60–61]. Preliminary data demonstrated that the highest prevalence of *B*. *burgdorferi* s. s. occurred in chipmunks in younger forest units (brushy pole and young multi-storied forests) and the highest prevalence of *B*. *bissettiae* was in woodrats occupying young multi-storied forests and non-forested grassland (G. M. Hacker, unpubl. data). A longer-term sampling effort within these forest units is needed to bolster sample sizes and better evaluate the preliminary habitat associations observed in this study. The abundance and distribution of *I*. *pacificus* and *I*. *spinipalpis* immatures within these forest units is also undoubtedly contributing to the variation observed in prevalence of *B*. *burgdorferi* s. l. in woodrats and chipmunks and offer fertile ground for future research.

## Conclusions

This is the first study demonstrating the importance of Allen’s chipmunks in the ecology of *B*. *burgdorferi* s. l. in the western United States. The spatial clustering of *B*. *burgdorferi* s. l. observed in this study can be explained, in part, by the spatial and temporal distribution of host species, time-of-year, and elevation. Spatial clustering and environmental associations of *B*. *burgdorferi* s. l. in vector ticks as well as in rodents should be evaluated in other forested areas of northern California to validate our findings and to clarify the vector-pathogen-host interrelationships. Most importantly, the public generally and foresters in particular should be informed about the potential risk of exposure to spirochete-infected vector ticks in certain subtypes of forests that harbor significant populations of woodrats and chipmunks.

## Supporting information

S1 Table All host-related information used for statistical analyses in this study. Data include all relevant host species measurements, date of capture, location of capture (UTM coordinates), related forest structure attributes, elevation, and PCR test results.
(PDF)

S2 Table All parameter coefficients, associated standard errors, and p-values for the top six models ranked via AICc. (PDF)

S3 Table Summary information on all multinomial log-linear models analyzed including the number of parameters, log likelihood, AICc score, delta AIC, model weight, and cumulative weight. (PDF)

S4 Table Sample ID, identification, and percent similarity to known GenBank isolates for all samples used for this study. (PDF)
