# Supplementary material for: Spatial clustering of Borrelia burgdorferi sensu lato within populations of Allen's chipmunks and dusky-footed woodrats in northwestern California
Source: PLoS One. 2018 Apr 10;13(4):e0195586. doi: 10.1371/journal.pone.0195586 (PMC5892934; doi:10.1371/journal.pone.0195586)
Supplement: S1 Table — Data include all relevant host species measurements, date of capture, location of capture (UTM coordinates), related forest structure attributes, elevation, and PCR test results. (PDF) [file pone.0195586.s001.pdf]

Sample ID	Date	PCR Result	Forest Unit	Area of Forest Unit (Meters^2)	Dominant Understory Vegetation	Dominant Overstory Vegetation	Host Species	Host Sex	Host Age	Host Weight (grams)	Northing	Easting	Elevation (meters)
1	6/8/2004	None	Old Forest	101623	Hardwood	Douglas Fir	Neotoma fuscipes	Female	Adult	218	4541870	445863	175
2	6/17/2004	None	Non-Forested	76931	Grass	Brush	Otospermophilus beecheyi	Male	Adult	820	4543300	444043	99
3	6/17/2004	None	Non-Forested	76931	Grass	Brush	Otospermophilus beecheyi	Female	Subadult	390	4543377	444064	100
4	6/17/2004	None	Non-Forested	89908	Grass	Brush	Otospermophilus beecheyi	Female	Adult	600	4543160	443973	98
5	6/18/2004	None	Non-Forested	76931	Grass	Brush	Otospermophilus beecheyi	Male	Adult	595	4543377	444023	101
6	6/18/2004	None	Non-Forested	89908	Grass	Brush	Otospermophilus beecheyi	Female	Adult	595	4543328	443921	101
7	6/18/2004	None	Non-Forested	89909	Grass	Brush	Otospermophilus beecheyi	Male	Adult	655	4543430	443842	101
8	6/18/2004	None	Non-Forested	76931	Grass	Brush	Otospermophilus beecheyi	Female	Adult	442	4543424	443981	103
9	6/18/2004	None	Non-Forested	179176	Grass	Brush	Otospermophilus beecheyi	Female	Adult	535	4543424	443960	103
10	6/18/2004	None	Non-Forested	89908	Grass	Brush	Otospermophilus beecheyi	Female	Adult	575	4543405	443861	101
11	6/18/2004	None	Non-Forested	89909	Grass	Brush	Otospermophilus beecheyi	Male	Adult	725	4543249	443942	100
12	6/18/2004	None	Non-Forested	76931	Grass	Brush	Otospermophilus beecheyi	Male	Adult	625	4543387	444037	101
13	6/23/2004	None	Old Forest	59735	Hardwood	Douglas Fir	Neotoma fuscipes	Female	Adult	195	4544193	446676	668
14	6/27/2004	None	Non-Forested	16662	Grass	Brush	Otospermophilus beecheyi	Female	Adult	515	4543462	443818	100
15	6/27/2004	None	Non-Forested	16663	Grass	Brush	Otospermophilus beecheyi	Female	Adult	495	4543442	443840	101
16	6/28/2004	Bbss	Old Forest	483294	Hardwood	Douglas Fir	Neotoma fuscipes	Female	Adult	170	4545692	445512	807
17	6/28/2004	Bbss	Old Forest	390235	Hardwood	Douglas Fir	Neotoma fuscipes	Female	Adult	240	4545635	445787	821
18	6/28/2004	None	Stem Exclusion (UR)	59786	Hardwood	Douglas Fir	Neotamias senex	Male	Adult	74	4548119	446852	614
19	6/28/2004	None	Stem Exclusion (UR)	59786	Hardwood	Douglas Fir	Neotamias senex	Female	Subadult	57	4548119	446852	614
20	6/29/2004	None	Old Forest	390235	Hardwood	Douglas Fir	Neotoma fuscipes	Female	Juvenile	135	4545588	445824	799
21	6/29/2004	None	Stem Exclusion (UR)	59786	Hardwood	Douglas Fir	Neotamias senex	Male	Subadult	64	4548119	446852	614
22	6/30/2004	None	Old Forest	390235	Hardwood	Douglas Fir	Neotamias senex	Male	Adult	106	4545578	445883	788
23	6/30/2004	None	Old Forest	390235	Hardwood	Douglas Fir	Neotamias senex	Female	Adult	100	4545501	445862	772
24	6/30/2004	None	Old Forest	390235	Hardwood	Douglas Fir	Neotamias senex	Female	Juvenile	55	4545578	445883	788
25	6/30/2004	None	Old Forest	390235	Hardwood	Douglas Fir	Neotamias senex	Female	Juvenile	58	4545578	445883	788
26	6/30/2004	None	Old Forest	52824	Hardwood	Douglas Fir	Neotamias senex	Female	Juvenile	60	4544734	446545	750
27	6/30/2004	Bbss	Old Forest	27926	Hardwood	Douglas Fir	Neotamias senex	Male	Adult	80	4544698	446537	756
28	7/1/2004	Bbss	Old Forest	52824	Hardwood	Douglas Fir	Neotamias senex	Male	Adult	90	4544718	446549	756
29	7/1/2004	None	Old Forest	52824	Hardwood	Douglas Fir	Neotamias senex	Male	Juvenile	52	4544773	446585	754
30	7/1/2004	None	Old Forest	27927	Hardwood	Douglas Fir	Neotamias senex	Male	Juvenile	54	4544698	446538	756
31	7/1/2004	None	Old Forest	52824	Hardwood	Douglas Fir	Neotamias senex	Female	Juvenile	54	4544690	446505	746
32	7/8/2004	Bbss	Old Forest	18240	Hardwood	Douglas Fir	Neotamias senex	Female	Juvenile	84	4545630	447332	956
33	7/8/2004	None	Stem Exclusion	96653	Douglas Fir	Tan Oak	Neotoma fuscipes	Male	Adult	280	4545610	447296	962
34	7/8/2004	None	Brushy Pole	13779	Brush	Douglas Fir	Neotoma fuscipes	Female	Juvenile	120	4545559	447264	964
35	7/8/2004	None	Brushy Pole	170314	Brush	Douglas Fir	Neotoma fuscipes	Female	Adult	240	4545516	447306	949
36	7/8/2004	Bbss	Brushy Pole	170314	Brush	Douglas Fir	Neotoma fuscipes	Male	Adult	280	4545429	447300	953
37	7/8/2004	None	Brushy Pole	170314	Brush	Douglas Fir	Neotoma fuscipes	Male	Adult	280	4545389	447332	945

38	7/8/2004	None	Brushy Pole	170314	Brush	Douglas Fir	Otospermophilus beecheyi	Female	Adult	490	4545415	447309	950
39	7/9/2004	None	Brushy Pole	170314	Brush	Douglas Fir	Neotoma fuscipes	Male	Adult	350	4545455	447309	950
40	7/9/2004	None	Brushy Pole	170314	Brush	Douglas Fir	Neotoma fuscipes	Female	Adult	280	4545485	447295	955
41	7/9/2004	None	Stem Exclusion	96653	Douglas Fir	Tan Oak	Neotoma fuscipes	Female	Adult	220	4545650	447322	961
42	7/9/2004	Bbss	Stem Exclusion	96653	Douglas Fir	Tan Oak	Neotamias senex	Female	Juvenile	74	4545650	447322	961
43	7/13/2004	None	Young Multistoried Forest	46158	Tan Oak	Douglas Fir	Neotoma fuscipes	Male	Subadult	140	4549865	435218	583
44	7/13/2004	None	Young Multistoried Forest	46158	Tan Oak	Douglas Fir	Neotoma fuscipes	Male	Subadult	170	4549787	435182	575
45	7/13/2004	Bbis	Young Multistoried Forest	46158	Tan Oak	Douglas Fir	Neotoma fuscipes	Male	Adult	320	4549773	435188	576
46	7/13/2004	Bbis	Brushy Pole	128062	Brush	Douglas Fir	Neotoma fuscipes	Male	Adult	290	4549731	435207	584
47	7/13/2004	None	Young Multistoried Forest	200200	Tan Oak	Douglas Fir	Neotoma fuscipes	Male	Adult	340	4549694	435202	584
48	7/13/2004	Bbis	Young Multistoried Forest	200200	Tan Oak	Douglas Fir	Neotoma fuscipes	Female	Adult	270	4549698	435204	588
49	7/13/2004	None	Young Multistoried Forest	200200	Tan Oak	Douglas Fir	Neotoma fuscipes	Female	Adult	230	4549647	435206	601
50	7/13/2004	None	Young Multistoried Forest	200200	Tan Oak	Douglas Fir	Neotoma fuscipes	Male	Subadult	140	4549627	435209	606
51	7/13/2004	None	Young Multistoried Forest	200200	Tan Oak	Douglas Fir	Neotoma fuscipes	Male	Subadult	150	4549614	435205	608
52	7/13/2004	Bbis	Young Multistoried Forest	200200	Tan Oak	Douglas Fir	Neotoma fuscipes	Male	Adult	310	4549615	435209	608
53	7/14/2004	Bbis	Young Multistoried Forest	46158	Tan Oak	Douglas Fir	Neotoma fuscipes	Female	Subadult	130	4549888	435254	598
54	7/14/2004	None	Young Multistoried Forest	46158	Tan Oak	Douglas Fir	Neotoma fuscipes	Male	Subadult	100	4549847	435202	579
55	7/14/2004	None	Young Multistoried Forest	46158	Tan Oak	Douglas Fir	Neotoma fuscipes	Male	Juvenile	90	4549815	435205	582
56	7/14/2004	None	Young Multistoried Forest	46158	Tan Oak	Douglas Fir	Neotoma fuscipes	Male	Adult	180	4549821	435201	580
57	7/14/2004	None	Young Multistoried Forest	46158	Tan Oak	Douglas Fir	Neotoma fuscipes	Female	Adult	250	4549766	435221	589
58	7/14/2004	None	Young Multistoried Forest	46158	Tan Oak	Douglas Fir	Neotoma fuscipes	Male	Adult	280	4549764	435223	589
59	7/14/2004	None	Young Multistoried Forest	200200	Tan Oak	Douglas Fir	Neotoma fuscipes	Female	Adult	230	4549694	435202	584
60	7/14/2004	None	Young Multistoried Forest	200200	Tan Oak	Douglas Fir	Neotoma fuscipes	Male	Subadult	160	4549698	435204	588
61	7/14/2004	Bbis	Young Multistoried Forest	200200	Tan Oak	Douglas Fir	Neotoma fuscipes	Female	Adult	200	4549673	435207	591
62	7/14/2004	Bbsl	Young Multistoried Forest	200200	Tan Oak	Douglas Fir	Neotoma fuscipes	Male	Adult	330	4549614	435205	608
63	7/14/2004	None	Young Multistoried Forest	200200	Tan Oak	Douglas Fir	Neotoma fuscipes	Female	Adult	230	4549615	435209	608
64	7/14/2004	None	Young Multistoried Forest	46158	Tan Oak	Douglas Fir	Neotamias senex	Female	Adult	109	4549865	435215	583
65	7/15/2004	Bbis	Young Multistoried Forest	46158	Tan Oak	Douglas Fir	Neotoma fuscipes	Male	Adult	260	4549880	435246	595
66	7/15/2004	None	Young Multistoried Forest	200200	Tan Oak	Douglas Fir	Neotoma fuscipes	Male	Adult	200	4549169	435191	583
67	7/15/2004	None	Young Multistoried Forest	46158	Tan Oak	Douglas Fir	Neotamias senex	Male	Juvenile	70	4549848	435211	581
68	7/16/2004	Bbsl	Young Multistoried Forest	46158	Tan Oak	Douglas Fir	Neotoma fuscipes	Female	Adult	240	4549790	435181	575
69	7/16/2004	None	Young Multistoried Forest	46158	Tan Oak	Douglas Fir	Neotoma fuscipes	Female	Adult	250	4549773	435188	576
70	7/16/2004	Bbis	Young Multistoried Forest	46158	Tan Oak	Douglas Fir	Neotamias senex	Male	Adult	110	4549821	435211	582
71	7/16/2004	None	Young Multistoried Forest	46158	Tan Oak	Douglas Fir	Neotamias senex	Female	Juvenile	70	4549916	435287	609
72	7/16/2004	None	Young Multistoried Forest	46158	Tan Oak	Douglas Fir	Neotamias senex	Female	Juvenile	80	4549865	435218	583
73	7/16/2004	Bbis	Young Multistoried Forest	46158	Tan Oak	Douglas Fir	Neotamias senex	Female	Adult	102	4549798	435175	573
74	7/16/2004	None	Young Multistoried Forest	46158	Tan Oak	Douglas Fir	Neotamias senex	Female	Subadult	82	4549779	435196	578
75	7/16/2004	None	Young Multistoried Forest	46158	Tan Oak	Douglas Fir	Neotamias senex	Male	Juvenile	60	4549819	435206	582
76	7/20/2004	Bbis	Young Multistoried Forest	46158	Tan Oak	Douglas Fir	Neotoma fuscipes	Male	Subadult	122	4549934	435315	616
77	7/20/2004	None	Old Forest	444927	Hardwood	Douglas Fir	Neotoma fuscipes	Male	Subadult	112	4549002	435518	638
78	7/20/2004	None	Old Forest	444927	Hardwood	Douglas Fir	Neotamias senex	Female	Juvenile	64	4549085	435613	681

79	7/20/2004	None	Old Forest	444927	Hardwood	Douglas Fir	Neotamias senex	Male	Subadult	82	4549014	435512	639
80	7/21/2004	None	Old Forest	444927	Hardwood	Douglas Fir	Neotoma fuscipes	Female	Adult	215	4548921	435294	550
81	7/21/2004	None	Young Multistoried Forest	46158	Tan Oak	Douglas Fir	Neotoma fuscipes	Female	Adult	160	4549934	435315	616
82	7/21/2004	None	Old Forest	444927	Hardwood	Douglas Fir	Neotoma fuscipes	Female	Adult	175	4548975	435276	554
83	7/21/2004	Bbis	Old Forest	444927	Hardwood	Douglas Fir	Neotoma fuscipes	Female	Adult	205	4549227	435499	680
84	7/21/2004	None	Old Forest	444927	Hardwood	Douglas Fir	Neotoma fuscipes	Female	Adult	170	4549085	435613	681
85	7/21/2004	Bbis	Old Forest	444927	Hardwood	Douglas Fir	Neotoma fuscipes	Female	Adult	235	4549056	435626	680
86	7/21/2004	None	Old Forest	444927	Hardwood	Douglas Fir	Neotoma fuscipes	Male	Juvenile	130	4548961	435589	651
87	7/21/2004	Bbis	Young Multistoried Forest	444927	Hardwood	Douglas Fir	Neotoma fuscipes	Male	Adult	290	4549934	435315	616
88	7/21/2004	None	Old Forest	444927	Hardwood	Douglas Fir	Neotoma fuscipes	Male	Adult	190	4548940	435265	543
89	7/22/2004	Bbis	Old Forest	444927	Hardwood	Douglas Fir	Neotoma fuscipes	Male	Adult	290	4549085	435613	681
90	7/22/2004	Bbis	Young Multistoried Forest	46158	Tan Oak	Douglas Fir	Neotamias senex	Male	Subadult	78	4549934	435315	616
91	7/22/2004	None	Old Forest	444927	Hardwood	Douglas Fir	Neotamias senex	Female	Adult	73	4549029	435510	641
92	7/22/2004	Bbss	Old Forest	444927	Hardwood	Douglas Fir	Neotamias senex	Female	Adult	100	4549075	435591	673
93	7/23/2004	None	Old Forest	444927	Hardwood	Douglas Fir	Neotoma fuscipes	Male	Adult	200	4548921	435294	550
94	7/23/2004	None	Old Forest	444927	Hardwood	Douglas Fir	Neotoma fuscipes	Male	Adult	240	4549010	435213	542
95	7/23/2004	Bbis	Young Multistoried Forest	46158	Tan Oak	Douglas Fir	Neotoma fuscipes	Female	Adult	240	4549921	435329	618
96	7/23/2004	None	Old Forest	444927	Hardwood	Douglas Fir	Neotamias senex	Male	Subadult	72	4549005	435579	656
97	7/23/2004	None	Old Forest	444927	Hardwood	Douglas Fir	Neotamias senex	Female	Adult	84	4548977	435237	542
98	7/25/2004	None	Old Forest	444927	Hardwood	Douglas Fir	Neotoma fuscipes	Male	Adult	320	4548994	435210	537
99	7/25/2004	None	Young Multistoried Forest	46158	Tan Oak	Douglas Fir	Neotoma fuscipes	Female	Adult	150	4549934	435315	616
100	7/25/2004	None	Old Forest	444927	Hardwood	Douglas Fir	Neotamias senex	Female	Subadult	84	4548977	435237	542
102	7/25/2004	None	Old Forest	444927	Hardwood	Douglas Fir	Neotamias senex	Female	Subadult	74	4549208	435480	675
103	7/26/2004	None	Old Forest	444927	Hardwood	Douglas Fir	Glaucomys sabrinus	Male	Adult	80	4549035	435542	652
104	7/26/2004	Bbis	Young Multistoried Forest	46158	Tan Oak	Douglas Fir	Neotamias senex	Female	Adult	122	4549934	435315	616
105	7/26/2004	None	Old Forest	444927	Hardwood	Douglas Fir	Neotamias senex	Male	Subadult	76	4549215	435512	681
106	7/26/2004	None	Old Forest	444927	Hardwood	Douglas Fir	Neotamias senex	Male	Subadult	80	4549227	435499	680
107	7/27/2004	Bbis	Old Forest	444927	Hardwood	Douglas Fir	Neotoma fuscipes	Female	Adult	250	4549004	435158	514
108	7/27/2004	None	Old Forest	444927	Hardwood	Douglas Fir	Neotamias senex	Female	Subadult	80	4549075	435591	673
109	7/28/2004	None	Old Forest	444927	Hardwood	Douglas Fir	Glaucomys sabrinus	Female	Subadult	144	4549057	435497	644
110	7/28/2004	Bbss	Old Forest	444927	Hardwood	Douglas Fir	Neotamias senex	Female	Subadult	92	4549014	435512	639
111	7/28/2004	Bbss	Old Forest	444927	Hardwood	Douglas Fir	Neotamias senex	Female	Adult	84	4548995	435614	663
112	7/28/2004	None	Old Forest	444927	Hardwood	Douglas Fir	Neotamias senex	Male	Subadult	60	4549227	435530	686
113	7/29/2004	None	Old Forest	444927	Hardwood	Douglas Fir	Neotoma fuscipes	Male	Adult	350	4548929	435259	542
114	7/29/2004	None	Old Forest	444927	Hardwood	Douglas Fir	Neotamias senex	Male	Subadult	74	4549063	435236	561
115	7/29/2004	None	Old Forest	444927	Hardwood	Douglas Fir	Neotamias senex	Male	Subadult	80	4548957	435225	534
116	7/29/2004	None	Old Forest	444927	Hardwood	Douglas Fir	Neotamias senex	Female	Subadult	92	4548955	435237	539
117	8/3/2004	None	Young Multistoried Forest	46158	Tan Oak	Douglas Fir	Glaucomys sabrinus	Female	Adult	120	4549954	435282	603
118	8/3/2004	None	Old Forest	444927	Hardwood	Douglas Fir	Glaucomys sabrinus	Female	Subadult	80	4549066	435538	656
119	8/3/2004	None	Old Forest	444927	Hardwood	Douglas Fir	Glaucomys sabrinus	Male	Adult	112	4549201	435529	683
120	8/3/2004	None	Young Multistoried Forest	46158	Tan Oak	Douglas Fir	Neotamias senex	Female	Subadult	84	4549981	435314	611

121	8/3/2004	None	Old Forest	444927	Hardwood	Douglas Fir	Neotamias senex	Male	Adult	76	4549183	435519	677
122	8/3/2004	None	Old Forest	444927	Hardwood	Douglas Fir	Neotamias senex	Male	Subadult	56	4549206	435506	678
123	8/3/2004	None	Old Forest	444927	Hardwood	Douglas Fir	Neotamias senex	Female	Subadult	72	4549209	435507	679
124	8/6/2004	None	Stem Exclusion (UR)	51432	Tan Oak	Douglas Fir	Neotoma fuscipes	Male	Adult	190	4549998	435278	601
125	8/6/2004	None	Stem Exclusion (UR)	51432	Tan Oak	Douglas Fir	Neotoma fuscipes	Male	Subadult	110	4550029	435225	581
126	8/6/2004	None	Old Forest	444927	Hardwood	Douglas Fir	Glaucomys sabrinus	Male	Adult	100	4549035	435542	652
127	8/6/2004	None	Old Forest	444927	Hardwood	Douglas Fir	Neotamias senex	Female	Subadult	72	4549218	435517	683
128	8/6/2004	Bbss	Old Forest	444927	Hardwood	Douglas Fir	Neotamias senex	Male	Subadult	78	4549002	435518	638
129	8/9/2004	None	Non-Forested	489523	Grass	Brush	Otospermophilus beecheyi	Male	Adult	595	4552603	439849	623
130	8/10/2004	None	Stem Exclusion (UR)	51432	Tan Oak	Douglas Fir	Neotamias senex	Male	Subadult	54	4550008	435240	587
131	8/10/2004	None	Stem Exclusion (UR)	51432	Tan Oak	Douglas Fir	Neotamias senex	Male	Subadult	56	4549978	435199	575
132	8/10/2004	None	Young Multistoried Forest	46158	Tan Oak	Douglas Fir	Neotamias senex	Female	Subadult	94	4549956	435295	608
133	8/10/2004	None	Stem Exclusion (UR)	51432	Tan Oak	Douglas Fir	Neotamias senex	Male	Subadult	50	4549998	435278	601
134	8/10/2004	None	Non-Forested	489523	Grass	Brush	Otospermophilus beecheyi	Male	Subadult	330	4552622	439839	625
135	8/10/2004	None	Non-Forested	489523	Grass	Brush	Otospermophilus beecheyi	Male	Subadult	350	4552630	439814	631
136	8/10/2004	None	Non-Forested	489523	Grass	Brush	Otospermophilus beecheyi	Male	Subadult	310	4552626	439824	628
137	8/10/2004	None	Non-Forested	489523	Grass	Brush	Otospermophilus beecheyi	Female	Adult	545	4552520	439922	615
138	8/10/2004	None	Non-Forested	489523	Grass	Brush	Otospermophilus beecheyi	Male	Subadult	455	4552843	439640	643
139	8/11/2004	None	Non-Forested	489523	Grass	Brush	Otospermophilus beecheyi	Female	Adult	470	4552843	439640	643
140	8/11/2004	None	Non-Forested	489523	Grass	Brush	Otospermophilus beecheyi	Female	Adult	535	4552622	439839	625
141	8/11/2004	None	Non-Forested	489523	Grass	Brush	Otospermophilus beecheyi	Female	Subadult	300	4552447	439881	614
142	8/11/2004	None	Non-Forested	489523	Grass	Brush	Otospermophilus beecheyi	Female	Subadult	290	4552825	439645	643
143	8/11/2004	None	Non-Forested	489523	Grass	Brush	Otospermophilus beecheyi	Female	Subadult	370	4552757	439688	638
144	8/12/2004	None	Non-Forested	489523	Grass	Brush	Otospermophilus beecheyi	Female	Adult	275	4552622	439839	625
145	8/17/2004	None	Brushy Pole	170314	Brush	Hardwood	Neotoma fuscipes	Male	Adult	310	4545262	447441	918
146	8/17/2004	None	Brushy Pole	139876	Hardwood	Douglas Fir	Neotoma fuscipes	Male	Adult	290	4545272	447396	931
147	8/17/2004	None	Brushy Pole	139876	Hardwood	Douglas Fir	Neotoma fuscipes	Male	Subadult	150	4545273	447393	931
148	8/17/2004	None	Brushy Pole	139876	Hardwood	Douglas Fir	Neotoma fuscipes	Female	Adult	200	4545259	447420	923
149	8/17/2004	None	Brushy Pole	170314	Brush	Hardwood	Neotoma fuscipes	Male	Adult	260	4545356	447355	941
150	8/17/2004	Bbss	Brushy Pole	170314	Brush	Hardwood	Neotoma fuscipes	Female	Adult	215	4545348	447348	943
151	8/17/2004	None	Brushy Pole	170314	Brush	Hardwood	Neotoma fuscipes	Female	Adult	260	4545421	447301	953
152	8/17/2004	None	Old Forest	483294	Hardwood	Douglas Fir	Neotoma fuscipes	Male	Adult	250	4545579	445411	841
153	8/17/2004	None	Old Forest	115443	Hardwood	Douglas Fir	Otospermophilus beecheyi	Female	Subadult	280	4545415	447210	973
154	8/17/2004	Bbss	Brushy Pole	170314	Brush	Hardwood	Neotamias senex	Male	Subadult	82	4545417	447301	953
155	8/18/2004	None	Brushy Pole	170314	Brush	Hardwood	Neotoma fuscipes	Male	Juvenile	50	4545356	447355	941
156	8/18/2004	None	Brushy Pole	170314	Brush	Hardwood	Neotoma fuscipes	Female	Adult	240	4545262	447441	918
157	8/18/2004	None	Brushy Pole	139876	Hardwood	Douglas Fir	Neotoma fuscipes	Female	Adult	220	4545273	447393	931
158	8/18/2004	None	Old Forest	483294	Hardwood	Douglas Fir	Neotoma fuscipes	Female	Adult	220	4545516	445388	832
160	8/18/2004	None	Brushy Pole	170314	Brush	Hardwood	Otospermophilus beecheyi	Female	Juvenile	220	4545415	447310	950
161	8/18/2004	None	Brushy Pole	170314	Brush	Hardwood	Neotamias senex	Female	Subadult	72	4545519	447275	959
162	8/18/2004	Bbss	Brushy Pole	170314	Brush	Hardwood	Neotamias senex	Female	Subadult	88	4545247	447441	919

163	8/18/2004	None	Brushy Pole	170314	Brush	Hardwood	Otospermophilus beecheyi	Female	Subadult	230	4545522	447280	959
164	8/18/2004	None	Brushy Pole	170314	Brush	Hardwood	Otospermophilus beecheyi	Female	Subadult	270	4545417	447301	953
165	8/20/2004	None	Old Forest	483294	Hardwood	Douglas Fir	Neotoma fuscipes	Female	Adult	155	4545516	445388	832
166	8/20/2004	None	Brushy Pole	170314	Brush	Hardwood	Neotoma fuscipes	Male	Adult	280	4545421	447301	953
167	8/20/2004	None	Brushy Pole	170314	Brush	Hardwood	Otospermophilus beecheyi	Male	Subadult	550	4545522	447280	959
168	8/20/2004	None	Brushy Pole	170314	Brush	Hardwood	Neotamias senex	Female	Adult	96	4545262	447441	918
169	8/20/2004	Bbss	Stem Exclusion	16099	Hardwood	Tan Oak	Neotamias senex	Female	Subadult	79	4545345	447344	943
170	8/20/2004	None	Brushy Pole	170314	Brush	Hardwood	Otospermophilus beecheyi	Female	Subadult	300	4545519	447275	959
171	8/20/2004	None	Brushy Pole	170314	Brush	Hardwood	Otospermophilus beecheyi	Female	Subadult	215	4545518	447277	959
172	1/18/2005	None	Oak Woodland	123453	Grass	Tan Oak	Glaucomys sabrinus	Male	Adult	90	4541599	446262	108
175	2/17/2005	None	Stem Exclusion	135631	Tan Oak	Hardwood	Neotoma fuscipes	Female	Subadult	135	4549406	447183	823
176	2/24/2005	None	Stem Exclusion	55426	Douglas Fir	Tan Oak	Neotoma fuscipes	Male	Adult	235	4554881	438728	845
177	2/27/2005	None	Non-Forested	11081	Grass	Brush	Neotoma fuscipes	Male	Adult	215	4553506	438450	758
180	3/1/2005	None	Brushy Pole	51985	Brush	Douglas Fir	Neotoma fuscipes	Male	Adult	275	4556682	438645	933
181	3/2/2005	None	Old Forest	12849	Hardwood	Douglas Fir	Neotamias senex	Male	Adult	85	4555485	438936	881
182	3/3/2005	None	Stem Exclusion	147977	Hardwood	Tan Oak	Neotoma fuscipes	Male	Adult	230	4556303	438957	918
183	3/3/2005	None	Stem Exclusion	147977	Hardwood	Tan Oak	Neotoma fuscipes	Female	Adult	215	4556273	438954	912
184	3/3/2005	None	Stem Exclusion	147977	Hardwood	Tan Oak	Neotoma fuscipes	Male	Adult	320	4556254	438931	915
185	3/3/2005	Bbis	Stem Exclusion	147977	Hardwood	Tan Oak	Neotoma fuscipes	Male	Subadult	255	4556120	438906	911
186	3/9/2005	None	Non-Forested	1033844	Grass	Brush	Otospermophilus beecheyi	Male	Adult	580	4552326	439930	597
187	3/10/2005	Bbis	Non-Forested	1033844	Grass	Brush	Neotoma fuscipes	Male	Adult	270	4552176	440105	558
188	3/10/2005	None	Non-Forested	1033844	Grass	Brush	Neotoma fuscipes	Female	Adult	180	4552168	439949	569
189	3/10/2005	Bbis	Non-Forested	1033844	Grass	Brush	Neotoma fuscipes	Male	Adult	375	4552326	439930	597
190	3/10/2005	Bbsl	Non-Forested	1033844	Grass	Brush	Neotoma fuscipes	Male	Adult	325	4552372	439875	606
191	3/10/2005	Bbis	Stem Exclusion	147977	Hardwood	Tan Oak	Neotoma fuscipes	Male	Adult	330	4556400	438999	924
192	3/10/2005	None	Stem Exclusion	93256	Douglas Fir	Tan Oak	Neotoma fuscipes	Female	Subadult	145	4556270	438950	913
193	3/10/2005	None	Non-Forested	1033844	Grass	Brush	Otospermophilus beecheyi	Male	Adult	540	4552368	439862	606
194	3/10/2005	None	Stem Exclusion	147977	Hardwood	Tan Oak	Neotamias senex	Female	Subadult	88	4556181	438885	917
195	3/11/2005	None	Stem Exclusion	93256	Douglas Fir	Tan Oak	Neotoma fuscipes	Female	Adult	190	4556026	438945	909
196	3/11/2005	None	Stem Exclusion	93256	Douglas Fir	Tan Oak	Neotoma fuscipes	Male	Adult	260	4556015	438942	912
197	3/11/2005	Bbsl	Non-Forested	1033844	Grass	Brush	Neotoma fuscipes	Female	Adult	235	4552368	439862	606
198	3/11/2005	None	Non-Forested	1033844	Grass	Brush	Neotoma fuscipes	Male	Adult	230	4552370	439873	606
199	3/11/2005	None	Non-Forested	1033844	Grass	Brush	Otospermophilus beecheyi	Male	Adult	775	4552359	439855	605
200	3/23/2005	None	Non-Forested	106570	Grass	Brush	Otospermophilus beecheyi	Female	Adult	530	4543453	443843	101
201	3/23/2005	None	Non-Forested	106570	Grass	Brush	Otospermophilus beecheyi	Male	Adult	600	4543457	443821	101
202	3/24/2005	None	Non-Forested	106570	Grass	Brush	Otospermophilus beecheyi	Male	Adult	810	4543464	443829	101
203	3/24/2005	None	Non-Forested	106570	Grass	Brush	Otospermophilus beecheyi	Male	Adult	765	4543469	443832	101
204	3/24/2005	None	Non-Forested	106570	Grass	Brush	Otospermophilus beecheyi	Female	Adult	475	4543453	443843	101
205	3/24/2005	None	Non-Forested	106570	Grass	Brush	Otospermophilus beecheyi	Female	Adult	490	4543457	443821	101
206	3/24/2005	None	Non-Forested	106570	Grass	Brush	Otospermophilus beecheyi	Male	Adult	610	4543411	443845	101
207	3/29/2005	None	Old Forest	12849	Hardwood	Douglas Fir	Neotoma fuscipes	Female	Adult	200	4555427	438955	872

208	3/29/2005	None	Old Forest	12849	Hardwood	Douglas Fir	Neotamias senex	Female	Adult	85	4555364	438927	865
209	3/29/2005	None	Old Forest	12849	Hardwood	Douglas Fir	Neotamias senex	Male	Adult	107	4555348	438949	856
210	3/30/2005	None	Old Forest	36831	Hardwood	Douglas Fir	Neotamias senex	Male	Adult	110	4555428	438871	886
211	3/30/2005	None	Old Forest	17388	Hardwood	Douglas Fir	Neotamias senex	Male	Adult	108	4555380	438872	878
212	3/30/2005	None	Non-Forested	1033844	Grass	Brush	Otospermophilus beecheyi	Male	Adult	640	4552800	439623	642
213	3/31/2005	Bbis	Non-Forested	1033844	Grass	Brush	Neotoma fuscipes	Female	Adult	300	4552907	439572	648
214	3/31/2005	Bbis	Non-Forested	1033844	Grass	Brush	Neotoma fuscipes	Female	Adult	195	4552774	439642	640
215	3/31/2005	None	Old Forest	17388	Hardwood	Douglas Fir	Neotamias senex	Male	Adult	97	4555380	438872	878
216	4/1/2005	Bbis	Non-Forested	1033844	Grass	Brush	Neotoma fuscipes	Female	Adult	245	4552908	439571	648
217	4/1/2005	None	Non-Forested	1033844	Grass	Brush	Neotoma fuscipes	Male	Adult	335	4552793	439601	641
218	4/1/2005	None	Non-Forested	1033844	Grass	Brush	Neotoma fuscipes	Female	Adult	190	4552810	439588	642
219	4/13/2005	None	Brushy Pole	238830	Brush	Tan Oak	Neotamias senex	Male	Adult	75	4549353	435476	687
220	4/13/2005	Bbis	Brushy Pole	238830	Brush	Tan Oak	Neotamias senex	Male	Adult	64	4549339	435498	689
222	4/13/2005	None	Brushy Pole	238830	Brush	Tan Oak	Neotoma fuscipes	Female	Adult	260	4549336	435475	687
223	4/13/2005	None	Brushy Pole	238830	Brush	Tan Oak	Neotoma fuscipes	Male	Adult	210	4549394	435454	681
224	4/13/2005	Bbis	Brushy Pole	238830	Brush	Tan Oak	Neotoma fuscipes	Female	Adult	250	4549340	435471	687
225	4/14/2005	None	Brushy Pole	238830	Brush	Tan Oak	Neotamias senex	Female	Adult	76	4549319	435509	689
226	4/14/2005	None	Brushy Pole	238830	Brush	Tan Oak	Neotamias senex	Female	Adult	85	4549339	435498	689
227	4/14/2005	None	Young Multistoried Forest	46158	Tan Oak	Douglas Fir	Neotamias senex	Female	Adult	80	4549888	435254	598
228	4/14/2005	None	Young Multistoried Forest	46158	Tan Oak	Douglas Fir	Neotamias senex	Male	Adult	84	4549847	435202	579
229	4/14/2005	Bbis	Brushy Pole	238830	Brush	Tan Oak	Neotamias senex	Male	Adult	68	4549363	435487	687
230	4/14/2005	None	Brushy Pole	238830	Brush	Tan Oak	Neotamias senex	Male	Adult	58	4549381	435428	679
231	4/14/2005	Bbss	Brushy Pole	238830	Brush	Tan Oak	Neotamias senex	Female	Adult	80	4549328	435466	687
232	4/15/2005	Bbis	Brushy Pole	238830	Brush	Tan Oak	Neotoma fuscipes	Female	Adult	275	4549381	435428	679
233	4/15/2005	Bbsl	Brushy Pole	238830	Brush	Tan Oak	Neotoma fuscipes	Female	Adult	245	4549431	435430	674
234	4/15/2005	None	Brushy Pole	238830	Brush	Tan Oak	Neotamias senex	Female	Adult	82	4549317	435511	689
235	4/15/2005	Bbis	Brushy Pole	238830	Brush	Tan Oak	Neotamias senex	Male	Adult	74	4549335	435504	689
236	4/20/2005	None	Brushy Pole	238830	Brush	Tan Oak	Neotoma fuscipes	Female	Adult	210	4544533	449168	437
237	4/20/2005	None	Stem Exclusion	131430	Brush	Hardwood	Neotoma fuscipes	Male	Adult	270	4544549	449162	443
238	4/21/2005	None	Brushy Pole	96064	Tan Oak	Douglas Fir	Neotoma fuscipes	Female	Adult	170	4544546	449290	447
239	4/21/2005	None	Stem Exclusion (UR)	58109	Tan Oak	Douglas Fir	Neotamias senex	Female	Adult	82	4544602	449136	457
240	4/22/2005	None	Stem Exclusion (UR)	58109	Tan Oak	Douglas Fir	Neotamias senex	Female	Adult	74	4544602	449136	457
241	4/22/2005	None	Brushy Pole	96064	Tan Oak	Douglas Fir	Neotoma fuscipes	Female	Adult	265	4544560	449305	451
242	4/22/2005	None	Stem Exclusion	131430	Tan Oak	Douglas Fir	Neotoma fuscipes	Female	Adult	230	4544514	449352	431
243	4/22/2005	None	Brushy Pole	96064	Tan Oak	Douglas Fir	Neotamias senex	Female	Adult	88	4544546	449290	447
244	4/23/2005	None	Oak Woodland	123453	Grass	Tan Oak	Otospermophilus beecheyi	Female	Adult	590	4541349	446495	130
245	4/23/2005	None	Oak Woodland	123453	Grass	Tan Oak	Otospermophilus beecheyi	Female	Adult	470	4541356	446529	122
246	4/23/2005	None	Oak Woodland	123453	Grass	Tan Oak	Otospermophilus beecheyi	Female	Adult	550	4541336	446517	125
247	4/23/2005	None	Oak Woodland	123453	Grass	Tan Oak	Otospermophilus beecheyi	Female	Adult	520	4541349	446495	130
248	4/25/2006	None	Oak Woodland	123453	Grass	Tan Oak	Otospermophilus beecheyi	Male	Adult	470	4541408	446533	122
249	4/25/2005	None	Oak Woodland	123453	Grass	Tan Oak	Otospermophilus beecheyi	Male	Adult	635	4541315	446476	134

250	4/25/2005	None	Oak Woodland	123453	Grass	Tan Oak	Otospermophilus beecheyi	Male	Adult	620	4541336	446517	125
251	4/25/2005	None	Oak Woodland	123453	Grass	Tan Oak	Otospermophilus beecheyi	Female	Adult	465	4541348	446494	130
252	4/25/2005	None	Oak Woodland	123453	Grass	Tan Oak	Otospermophilus beecheyi	Male	Adult	650	4541334	446495	130
253	4/25/2005	None	Oak Woodland	123453	Grass	Tan Oak	Otospermophilus beecheyi	Female	Adult	540	4541316	446499	128
254	4/25/2005	None	Oak Woodland	123453	Grass	Tan Oak	Otospermophilus beecheyi	Female	Subadult	335	4541428	446524	122
255	4/25/2005	None	Oak Woodland	123453	Grass	Tan Oak	Otospermophilus beecheyi	Male	Adult	590	4541398	446535	122
256	4/25/2005	None	Oak Woodland	123453	Grass	Tan Oak	Otospermophilus beecheyi	Female	Adult	440	4541420	446497	126
257	4/25/2005	None	Oak Woodland	123453	Grass	Tan Oak	Otospermophilus beecheyi	Female	Adult	710	4541326	446502	128
258	4/26/2005	None	Oak Woodland	123453	Grass	Tan Oak	Otospermophilus beecheyi	Female	Adult	560	4541488	446230	130
259	4/26/2005	None	Oak Woodland	123453	Grass	Tan Oak	Otospermophilus beecheyi	Male	Adult	505	4541431	446288	140
260	4/26/2005	None	Oak Woodland	123453	Grass	Tan Oak	Otospermophilus beecheyi	Male	Adult	510	4541373	446409	140
261	4/26/2005	None	Oak Woodland	123453	Grass	Tan Oak	Otospermophilus beecheyi	Male	Adult	470	4541404	446399	138
262	4/26/2005	None	Oak Woodland	123453	Grass	Tan Oak	Otospermophilus beecheyi	Female	Adult	415	4541319	446444	140
263	4/26/2005	None	Oak Woodland	123453	Grass	Tan Oak	Otospermophilus beecheyi	Female	Adult	580	4541336	446517	125
264	4/27/2005	None	Oak Woodland	123453	Grass	Tan Oak	Otospermophilus beecheyi	Female	Adult	410	4541464	446254	135
265	5/10/2005	None	Brushy Pole	150996	Brush	Douglas Fir	Neotoma fuscipes	Female	Adult	238	4545658	450821	932
266	5/10/2005	None	Brushy Pole	150996	Brush	Douglas Fir	Neotoma fuscipes	Male	Adult	253	4545601	450729	925
267	5/10/2005	None	Brushy Pole	150996	Brush	Douglas Fir	Neotoma fuscipes	Male	Adult	338	4545577	450686	923
268	5/10/2005	None	Brushy Pole	5959	Douglas Fir	Brush	Otospermophilus beecheyi	Female	Adult	510	4546647	449289	892
269	5/11/2005	None	Brushy Pole	150996	Brush	Douglas Fir	Neotoma fuscipes	Female	Adult	265	4545658	450821	932
270	5/11/2005	None	Brushy Pole	150996	Brush	Douglas Fir	Neotoma fuscipes	Female	Adult	190	4545627	450782	925
271	5/11/2005	None	Brushy Pole	150996	Brush	Douglas Fir	Neotoma fuscipes	Female	Adult	230	4545609	450763	920
272	5/11/2005	None	Brushy Pole	150996	Brush	Douglas Fir	Neotoma fuscipes	Female	Adult	220	4545577	450686	923
273	5/11/2005	Bbss	Stem Exclusion	137924	Hardwood	Tan Oak	Neotamias senex	Female	Adult	102	4546597	449264	886
274	5/11/2005	None	Brushy Pole	5959	Douglas Fir	Brush	Otospermophilus beecheyi	Female	Subadult	420	4546647	449289	892
275	5/11/2005	None	Brushy Pole	5959	Douglas Fir	Brush	Otospermophilus beecheyi	Female	Subadult	460	4546657	449289	891
276	5/11/2005	None	Brushy Pole	5959	Douglas Fir	Brush	Otospermophilus beecheyi	Male	Adult	495	4546633	449228	887
277	5/11/2005	None	Brushy Pole	5959	Douglas Fir	Brush	Otospermophilus beecheyi	Male	Adult	565	4546665	449269	890
278	5/11/2005	None	Stem Exclusion	137924	Hardwood	Tan Oak	Otospermophilus beecheyi	Female	Subadult	455	4546634	449208	884
279	5/12/2005	None	Brushy Pole	150996	Brush	Douglas Fir	Neotoma fuscipes	Male	Adult	200	4545635	450793	925
280	5/12/2005	None	Brushy Pole	150996	Brush	Douglas Fir	Neotoma fuscipes	Male	Adult	200	4545627	450782	925
281	5/12/2005	None	Brushy Pole	150996	Brush	Douglas Fir	Neotoma fuscipes	Female	Adult	260	4545601	450729	925
282	5/12/2005	None	Brushy Pole	150996	Brush	Douglas Fir	Neotoma fuscipes	Female	Adult	195	4545577	450686	923
283	5/12/2005	None	Stem Exclusion	137924	Hardwood	Tan Oak	Neotoma fuscipes	Male	Adult	240	4546634	449208	884
284	5/12/2005	None	Young Multistoried Forest	897277	Douglas Fir	Douglas Fir	Otospermophilus beecheyi	Female	Subadult	390	4546657	449289	891
285	5/12/2005	None	Stem Exclusion	137924	Hardwood	Tan Oak	Otospermophilus beecheyi	Male	Adult	475	4546607	449216	881
286	5/12/2005	None	Stem Exclusion	137924	Hardwood	Tan Oak	Otospermophilus beecheyi	Female	Adult	580	4546634	449208	884
287	5/12/2005	Bbss	Brushy Pole	150996	Brush	Douglas Fir	Neotamias senex	Female	Adult	100	4545645	450818	926
288	5/18/2005	None	Old Forest	15993	Hardwood	Douglas Fir	Neotamias senex	Female	Adult	94	4549035	448036	902
289	5/19/2005	None	Stem Exclusion	538127	Hardwood	Tan Oak	Neotamias senex	Female	Adult	81	4549835	447780	854
290	5/19/2005	None	Stem Exclusion	538127	Hardwood	Tan Oak	Neotamias senex	Female	Adult	84	4549866	447778	854

291	5/20/2005	None	Stem Exclusion	538127	Hardwood	Tan Oak	Neotamias senex	Female	Adult	86	4549816	447809	848
