# Supplementary material for: Spatial clustering of Borrelia burgdorferi sensu lato within populations of Allen's chipmunks and dusky-footed woodrats in northwestern California
Source: PLoS One. 2018 Apr 10;13(4):e0195586. doi: 10.1371/journal.pone.0195586 (PMC5892934; doi:10.1371/journal.pone.0195586)
Supplement: S2 Table — (PDF) [file pone.0195586.s002.pdf]

Top Model	Species + Month + Elevation								
Coefficient	Intercept	Chipmunk	August	February	July	June	March	May	Elevation
Bbss	-11.9956149	2.5218973	-0.1347212	-9.937327	0.58379669	0.790721	-30.7799806	-1.058475	0.010724898
Bbis	0.9543247	-0.8696813	-18.4957073	-19.47645	-0.01421653	-15.025434	0.5258654	-19.848285	-0.002916104
Std. Error									
Bbss	0.6940213	0.5960176	1.25E+00	2.42E-05	1.0195524	1.18E+00	1.72E-12	1.41E+00	0.001969179
Bbis	1.2225606	0.4900423	2.57E-08	1.66E-09	0.5676015	6.32E-07	8.07E-01	5.38E-09	0.001958867
P value									
Bbss	0	2.324E-05	0.9143394	0	0.5669149	0.5012814	0	0.4524748	5.14053E-08
Bbis	0.4350407	0.07594632	0	0	0.9800177	0	0.514655	0	0.1365746

2nd Model	Species + Elevation		
Coefficient	Intercept	Chipmunk	Elevation
Bbss	-9.029762	2.1436335	0.007190432
Bbis	1.456805	-0.9647639	-0.004065411
Std. Error			
Bbss	2.0344246	0.6499732	0.002219642
Bbis	0.9771238	0.4701274	0.001444165
P-value			
Bbss	9.06E-06	0.000973644	0.001197584
Bbis	1.36E-01	0.040156758	0.00487683

3rd Model	Stand type + Species + Elevation							
Coefficient	Intercept	Non-Forested	Old Forest	Stem Exclusion	Stem Exclusion (UR)	Young Multistoried Forest	Chipmunk	Elevation
Bbss	-8.07123	-11.025038	0.2951524	-0.47457418	-16.94617	-28.3742379	1.9776193	0.00619335
Bbis	1.738053	1.059226	-1.4788528	0.04508512	-25.79994	0.3376628	-0.4341522	-0.004436056
Std. Error								
Bbss	2.427324	6.27E-06	0.7079437	0.9473914	1.25E-07	2.13E-12	0.7041096	0.00264012
Bbis	1.208902	7.46E-01	0.7402742	0.885324	1.47E-11	5.72E-01	0.5245418	0.001661469
P-value								
Bbss	0.000883693	0	0.67674053	0.6164223	0	0	0.004974493	0.018983254
Bbis	0.150515535	0.1556725	0.04574816	0.9593853	0	0.5552543	0.407852384	0.007586008

4th Model	Stand type + Species + Month + Elevation													
Coefficient	Intercept	Non-Forested	Old Forest	Stem Exclusion	Stem Exclusion (UR)	Young Multistoried Forest	Chipmunk	August	February	July	June	March	May	Elevation
Bbss	-12.3498502	-10.9798416	1.414382	0.3411667	-14.14127	-16.44528919	2.0887559	-1.043025	-29.29125	-0.6764573	-0.7848685	-52.2678651	-2.140963	0.0120948
Bbis	0.5012274	0.7809413	-1.651223	-0.5307496	-18.26412	-0.009395084	-0.3043488	-24.357797	-25.88231	0.4941208	-21.618833	0.7268806	-24.726267	-0.0024942
Std. Error														
Bbss	0.1048431	2.55E-07	0.6900538	1.138688	6.59E-08	1.26E-08	0.660271	6.21E-01	7.28E-14	0.481208	6.44E-01	3.54E-16	7.42E-01	0.000751758
Bbis	1.4329376	1.03E+00	0.8255693	1.150109	1.14E-08	6.76E-01	0.5836997	7.17E-11	1.01E-11	0.7340666	2.32E-10	1.02E+00	4.70E-11	0.00216876
P-value														
Bbss	0	0	0.04039678	0.7644716	0	0	0.001558935	0.09287135	0	0.1597989	0.2230426	0	0.003906225	0
Bbis	0.7264962	0.4484409	0.04548929	0.6444558	0	0.9889186	0.602078814	0	0	0.5008658	0	0.4761147	0	0.2501201

5th Model	Stand type + Elevation						
Coefficient	Intercept	Non-Forested	Old Forest	Stem Exclusion	Stem Exclusion (UR)	Young Multistoried Forest	Elevation
Bbss	-6.78639	-10.716267	1.194625	-0.005408787	-9.830403	-14.6603879	0.005445435
Bbis	1.720995	1.146731	-1.593387	0.061282927	-19.038795	0.2332486	-0.004547933
Std. Error							
Bbss	2.264051	1.53E-05	0.6617705	0.8783278	6.39E-05	1.14E-06	0.002484528
Bbis	1.219904	7.40E-01	0.7194342	0.8837248	1.57E-08	5.53E-01	0.001688086
P-value							
Bbss	0.002722448	0	0.07104413	0.9950866	0	0	0.028398401
Bbis	0.158314664	0.1211138	0.02677528	0.9447141	0	0.6733517	0.007057159

6th Model	Stand type + Area of Stand + Elevation							
Coefficient	Intercept	Non-Forested	Old Forest	Stem Exclusion	Stem Exclusion (UR)	Young Multistoried Forest	Area of Stand	Elevation
Bbss	-8.525651	-15.66058	1.004833	-0.04870291	-6.290654	-13.2283594	2.03E-06	0.007012587
Bbis	1.469759	-1.35126	-2.407161	-0.06979021	-15.29952	0.5299782	3.01E-06	-0.004885849
Std. Error								
Bbss	4.94E-07	9.74E-15	5.64E-08	1.00E-07	1.25E-11	2.86E-14	1.11E-06	0.000473046
Bbis	6.30E-07	8.65E-08	3.26E-08	6.68E-08	3.03E-14	2.98E-07	7.15E-07	0.000434107
P-value								
Bbss	0	0	0	0	0	0	6.87E-02	0
Bbis	0	0	0	0	0	0	2.63E-05	0
