# Supplementary material for: Spatial clustering of Borrelia burgdorferi sensu lato within populations of Allen's chipmunks and dusky-footed woodrats in northwestern California
Source: PLoS One. 2018 Apr 10;13(4):e0195586. doi: 10.1371/journal.pone.0195586 (PMC5892934; doi:10.1371/journal.pone.0195586)
Supplement: S3 Table — (PDF) [file pone.0195586.s003.pdf]

Model Name	Number of Parameters	Log Likelihood	AICc	Δ AIC	Model Weight
Species + Month+ Elevation	18	-106.66	253.14	0.00	0.84
Species + Elevation	6	-122.34	257.11	3.97	0.12
Forest Unit + Species + Elevation	16	-112.21	259.42	6.28	0.04
Forest Unit + Species + Month+ Elevation	28	-99.41	264.42	11.28	0.00
Forest Unit + Elevation	14	-117.46	265.21	12.07	0.00
Forest Unit + Area of Unit + Elevation	16	-115.85	266.71	13.57	0.00
Month + Elevation	16	-116.20	267.40	14.26	0.00
Forest Unit + Species	14	-119.83	269.96	16.82	0.00
Species + Month	16	-117.63	270.26	17.12	0.00
Forest Unit + Species + Month	26	-105.14	270.49	17.35	0.00
Elevation	4	-132.15	272.50	19.36	0.00
Dominant Overstory Veg. + Species	10	-126.20	273.58	20.44	0.00
Species + Weight	12	-123.99	273.66	20.52	0.00
Weight	12	-123.99	273.66	20.52	0.00
Dominant Understory Veg. + Species	14	-121.88	274.05	20.91	0.00
Forest Unit	12	-124.97	275.62	22.48	0.00
Species	4	-134.33	276.88	23.74	0.00
Species + Sex + Weight	14	-123.34	276.98	23.84	0.00
Month	14	-124.08	278.45	25.31	0.00
Species + Sex	6	-133.01	278.46	25.32	0.00
Forest Unit + Area of Unit	14	-124.12	278.53	25.39	0.00
Species + Sex + Weight + Species X Sex	16	-121.86	278.72	25.58	0.00
Forest Unit + Species + Forest Unit X Species	22	-114.64	279.06	25.92	0.00
Species + Age	8	-131.17	279.11	25.97	0.00
Forest Unit + Area of Unit + Dominant Overstory Veg.	20	-117.24	279.22	26.08	0.00
Dominant Understory Veg.	12	-126.97	279.62	26.48	0.00
Species + Sex + Age	10	-129.77	280.71	27.57	0.00
Dominant Overstory Veg.	8	-132.60	281.97	28.83	0.00
Null	2	-141.42	286.90	33.76	0.00
Forest Unit + Area of Unit + Dominant Understory Veg.	22	-119.16	288.11	34.97	0.00
Forest Unit + Area of Unit + Dominant Overstory Veg. + Dominant Understory Veg.	28	-113.18	291.98	38.84	0.00
Full = Forest Unit + Area of Unit + Dominant Overstory veg. + Dominant Understory Veg. + Species + Sex + Age + Weight + Month + Elevation	60	-69.89	313.20	60.06	0.00

Cumulative Weight
0.841
0.956
0.993
0.996
0.998
0.999
0.999
1.000
1.000
1.000
1.000
1.000
1.000
1.000
1.000
1.000
1.000
1.000
1.000
1.000
1.000
1.000
1.000
1.000
1.000
1.000
1.000
1.000
1.000
1.000
1.000
1.000
