# Supplementary material for: Spatial clustering of Borrelia burgdorferi sensu lato within populations of Allen's chipmunks and dusky-footed woodrats in northwestern California
Source: PLoS One. 2018 Apr 10;13(4):e0195586. doi: 10.1371/journal.pone.0195586 (PMC5892934; doi:10.1371/journal.pone.0195586)
Supplement: S4 Table — (PDF) [file pone.0195586.s004.pdf]

ID	Result	Percent Identity	Sequence
EPBBrown 16	*Borrelia burgdorferi* sensu stricto	100%	TGAGTAGGCTATTGCCAGGGTTTTTATTTTTATCTTCCATCTCTATTTTG<br>CCAATTTATTTATACAACATAAAATAATATATATCTTTGTTTAATGCATG<br>TCAATATATATATTTTATTTTTTATGTTATTTAAACAACACATTCAAAAA<br>CACCAATATTTAAAAAACATAAAAATAAAATCAAAGTTTAAAGTATAAAA<br>ATAAAAACCCTGGCAATAACCTACTCA
EPBBrown 17	*Borrelia burgdorferi* sensu stricto	100%	TATTTTTATGTTTTTTAAATATTGGTGTTTTTGAATGTGTTGTTTAAATA<br>ACATAAAAAATAAAATATATATTGACATGTATTAAACAAAGATATATATT<br>ATTTTATGTTGTATAAATAAATTGGCAAAATAGAGATGGAAGATAAAAAT<br>AAAAACCCTGGCAATAGCCTACTCA
EPBBrown 27	*Borrelia burgdorferi* sensu stricto	100%	TGAGTAGGTTATTGCCAGGGTTTTTATTTTTATACTTTAAACTTTGATTT<br>TATTTTTATGTTTTTTAAATATTGGTGTTTTTGAATGTGTTGTTTAAATA<br>ACATAAAAAATAAAATATATATTGACATGTATTAAACAAAGATATATATT<br>ATTTTATGTTGTATAAATAAATTGGCAAAATAGAGATGGAAGATAAAAAT<br>AAAAACCCTGG
EPBBrown 28	*Borrelia burgdorferi* sensu stricto	100%	TGAGTAGGTTATTGCCAGGGTTTTTATTTTTATACTTTAAACTTTGATTT<br>TATTTTTATGTTTTTTAAATATTGGTGTTTTTGAATGTGTTGTTTAAATA<br>ACATAAAAAATAAAATATATATATTGACATGCATTAAACAAAGATATATA<br>TTATTTTATGTTGTATAAATAAATTGGCAAAATAGAGATGGAAGATAAAA<br>ATAAAAACCC
EPBBrown 32	*Borrelia burgdorferi* sensu stricto	99%	TGAGTAGGTTATTGCCAGGGTTTTTATTTTTATACTTTAAACTTTGATTT<br>TATTTTTATGTTTTTTAAATATTGGTGTTTTTGAATGTGTTGTTTAAATA<br>ACATAAAAAATAAAATATATATATTGACATGCAATAAACAAAGATATATA<br>TTATTTTATGTTGTATAAATAAATTGGCAAAATAGAGATGGAAGATAAAA<br>ATAAAAACCCTGG
EPBBrown 36	*Borrelia burgdorferi* sensu stricto	100%	TGAGTAGGTTATTGCCAGGGTTTTTATTTTTATACTTTAAACTTTGATTT<br>TATTTTTATGTTTTTTAAATATTGGTGTTTTTGAATGTGTTGTTTAAATA<br>ACATAAAAAATAAAATATATATATTGACATGCATTAAACAAAGATATATA<br>TTATTTTATGTTGTATAAATAAATTGGCAAAATAGAGATGGAAGATAAAA<br>ATAAAAACGCTGGC
EPBBrown 42	*Borrelia burgdorferi* sensu stricto	99%	TGAGTAGGTTATTGCCAGGGTTTTTATTTTTATACTTTAAACTTTGATTT<br>TATTTTTATGTTTTTTAAATATTGGTGTTTTTGAATGTGTTGTTTAAATA<br>ACATAAAAAATAAAATATATATATTGACATGCATTAAACAAAGATATATA<br>TTATTTTAGTGTTGTATAAATAAATTGGCAAAATAGAGATGGAAGATAAA<br>AATAAAAACCCTGGC
EPBBrown 45	*Borrelia bissettiae*	100%	TGAGTAGGTTATTGCCAGGGTTTTTATTTTTATATTTTAAATCTTGATTT<br>TATTTTATGTTTTTTAAATGTTTGTTAGTTTTTTTGAATGTGTTATTTAA<br>ATAACATAAAAAATAAAATATATATTGACATGGATTAAACAAAGATATAT<br>ATTATTTTATGTTGCGTAAACAAATTGGCAAAATAGAGATGGAAGATAAA<br>AATAAAAACCCTGGCAATAACCTACTCA
EPBBrown 46	*Borrelia bissettiae*	100%	TGAGTAGGTTATTGCCAGGGTTTTTATTTTTATATTTTAAATCTTGATTT<br>TATTTTATGTTTTTTAAATGTTTGTTAGTTTTTTTGAATGTGTTATTTAA<br>ATAACATAAAAAATAAAATATATATTGACATGGATTAAACAAAGATATAT<br>ATTATTTTATGTTGCGTAAACAAATTGGCAAAATAGAGATGGAAGATAAA<br>AATAAAAACCCTGGCAATAACCTACTCA
EPBBrown 48	*Borrelia bissettiae*	100%	TGAGTAGGTTATTGCCAGGGTTTTTATTTTTATATTTTAAATCTTGATTT<br>TATTTTATGTTTTTTAAATGTTTGTTAGTTTTTTTGAATGTGTTATTTAA<br>ATAACATAAAAAATAAAATATATATTGACATGGATTAAACAAAGATATAT<br>ATTATTTTATGTTGCGTAAACAAATTGGCAAAATAGAGATGGAAGATAAA<br>AATAAAAACCCTGGCAATAACCTACTCA
EPBBrown 52	*Borrelia bissettiae*	100%	TGAGTAGGTTATTGCCAGGGTTTTTATTTTTATATTTTAAATCTTGATTT<br>TATTTTATGTTTTTTAAATGTTTGTTAGTTTTTTTGAATGTGTTATTTAA<br>ATAACATAAAAAATAAAATATATATTGACATGGATTAAACAAAGATATAT<br>ATTATTTTATGTTGCGTAAACAAATTGGCAAAATAGAGATGGAAGATAAA<br>AATAAAAACCCTGGCAATAACCTACTCA
EPBBrown 53	*Borrelia bissettiae*	100%	TGAGTAGGTTATTGCCAGGGTTTTTATTTTTATATTTTAAATCTTGATTT<br>TATTTTATGTTTTTTAAATGTTTGTTAGTTTTTTTGAATGTGTTATTTAA<br>ATAACATAAAAAATAAAATATATATTGACATGGATTAAACAAAGATATAT<br>ATTATTTTATGTTGCGTAAACAAATTGGCAAAATAGAGATGGAAGATAAA<br>AATAAAAACCCTGGCAATAACCTACTCA

EPBBrown 61	*Borrelia bissettiae*	100%	TGAGTAGGTTATTGCCAGGGTTTTTATTTTTATATTTTAAATCTTGATTT<br>TATTTTATGTTTTTTAAATGTTTGTTAGTTTTTTTGAATGTGTTATTTAA<br>ATAACATAAAAAAATAAAATATATATTGACATGGATTAAACAAAGATATAT<br>ATTATTTTATGTTGCGTAAACAAATTGGCAAAATAGAGATGGAAGATAAA<br>AATAAAAA
EPBBrown 62	*Borrelia burgdorferi sensu lato*	N/A	N/A No clean sequence
EPBBrown 65	*Borrelia bissettiae*	100%	TGAGTAGGTTATTGCCAGGGTTTTTATTTTTATATTTTAAATCTTGATTT<br>TATTTTATGTTTTTTAAATGTTTGTTAGTTTTTTTGAATGTGTTATTTAA<br>ATAACATAAAAAAATAAAATATATATTGACATGGATTAAACAAAGATATAT<br>ATTATTTTATGTTGCGTAAACAAATTGGCAAAATAGAGATGGAAGATAAA<br>AATAAAAA
EPBBrown 68	*Borrelia burgdorferi sensu lato*	N/A	N/A No clean sequence
EPBBrown 70	*Borrelia bissettiae*	100%	TGAGTAGGTTATTGCCAGGGTTTTTATTTTTATATTTTAAATCTTGATTT<br>TATTTTATGTTTTTTAAATGTTTGTTAGTTTTTTTGAATGTGTTATTTAA<br>ATAACATAAAAAAATAAAATATATATTGACATGGATTAAACAAAGATATAT<br>ATTATTTTATGTTGCGTAAACAAATTGGCAAAATAGAGATGGAAGATAAA<br>AATAAAAACCCTGGCAATAACCTACTCA
EPBBrown 73	*Borrelia bissettiae*	100%	TGAGTAGGTTATTGCCAGGGTTTTTATTTTTATATTTTAAATCTTGATTT<br>TATTTTATGTTTTTTAAATGTTTGTTAGTTTTTTTGAATGTGTTATTTAA<br>ATAACATAAAAAAATAAAATATATATTGACATGGATTAAACAAAGATATAT<br>ATTATTTTATGTTGCGTAAACAAATTGGCAAAATAGAGATGGAAGATAAA<br>AATAAAAACCCTGGCAATAACCTACTCA
EPBBrown 76	*Borrelia bissettiae*	100%	TGAGTAGGTTATTGCCAGGGTTTTTATTTTTATATTTTAAATCTTGATTT<br>TATTTTATGTTTTTTAAATGTTTGTTAGTTTTTTTGAATGTGTTATTTAA<br>ATAACATAAAAAAATAAAATATATATTGACATGGATTAAACAAAGATATAT<br>ATTATTTTATGTTGCGTAAACAAATTGGCAAAATAGAGATGGAAGATAAA<br>AATAAAAACCCTGGCAATAACCTACTCA
EPBBrown 83	*Borrelia bissettiae*	100%	TGAGTAGGTTATTGCCAGGGTTTTTATTTTTATATTTTTAATCTTGATTT<br>TATTTTTTATGTTATTTAAATAACATAAAAAAATAAAATATATATTGACAT<br>GGATTAAACAAAGATATATATTATTTTATGTTGTATAAACAAATTGGCAA<br>AATAGAGATGGAAGATAAAAATAAAAACCCTGGCAATAACCTACTCA
EPBBrown 85	*Borrelia bissettiae*	100%	TGAGTAGGTTATTGCCAGGGTTTTTATTTTTATATTTTAAATCTTGATTT<br>TATTTTATGTTTTTTAAATGTTAGTGTTTTTGAATATATTATTTAAATAA<br>CATAAAAAATAAAATATATATTGACATGGATTAAACAAAGATATATATTA<br>TTTTATGTTGCATAAACAAATTGGCAAAATAGAGATGGAAGATAAAAATA<br>AAAACCC
EPBBrown 87	*Borrelia bissettiae*	100%	TGAGTAGGTTATTGCCAGGGTTTTTATTTTTATATTTTAAATCTTGATTT<br>TATTTTATGTTTTTTAAATGTTTGTTAGTTTTTTTGAATGTGTTATTTAA<br>ATAACATAAAAAAATAAAATATATATTGACATGGATTAAACAAAGATATAT<br>ATTATTTTATGTTGCGTAAACAAATTGGCAAAATAGAGATGGAAGATAAA<br>AATAAAAACCCTGGCAATAACCTACTCA
EPBBrown 89	*Borrelia bissettiae*	100%	TGAGTAGGTTATTGCCAGGGTTTTTATTTTTATATTTTAAATCTTGATTT<br>TATTTTATGTTTTTTAAATGTTAGTGTTTTTGAATATATTATTTAAATAA<br>CATAAAAAATAAAATATATATTGACATGGATTAAACAAAGATATATATTA<br>TTTTATGTTGCATAAACAAATTGGCAAAATAGAGATGGAAGATAAAAATA<br>AAAACCCTGGCAATAACCTACTCA
EPBBrown 90	*Borrelia bissettiae*	100%	TGAGTAGGTTATTGCCAGGGTTTTTATTTTTATATTTTAAATCTTGATTT<br>TATTTTATGTTTTTTAAATGTTAGTGTTTTTGAATATATTATTTAAATAA<br>CATAAAAAATAAAATATATATTGACATGGATTAAACAAAGATATATATTA<br>TTTTATGTTGCATAAACAAATTGGCAAAATAGAGATGGAAGATAAAAATA<br>AAAACCC

EPBBrown 92	*Borrelia burgdorferi* sensu stricto	100%	TGAGTAGGTTATTGCCAGGGTTTTTATTTTTATACTTTAAACTTTGATTT<br>TATTTTTATGTTTTTTAAATATTGGTGTTTTTGAATGTGTTGTTTAAATA<br>ACATAAAAAATAAAATATATATATTGACATGCATTAAACAAAGATATATA<br>TTATTTTATGTTGTATAAATAAATTGGCAAAATAGAGATGGAAGATAAAA<br>ATAAAAACCC
EPBBrown 95	*Borrelia bissettiae*	100%	TGAGTAGGTTATTGCCAGGGTTTTTATTTTTATATTTTAAATCTTGATTT<br>TATTTTATGTTTTTTAAATGTTTGTTAGTTTTTTTGAATGTGTTATTTAA<br>ATAACATAAAAAATAAAATATATATTGACATGGATTAAACAAAGATATAT<br>ATTATTTTATGTTGCGTAAACAAATTGGCAAAATAGAGATGGAAGATAAA<br>AATAAAAA
EPBBrown 104	*Borrelia bissettiae*	100%	TGAGTAGGTTATTGCCAGGGTTTTTATTTTTATATTTTAAATCTTGATTT<br>TATTTTATGTTTTTTAAATGTTTGTTAGTTTTTTTGAATGTGTTATTTAA<br>ATAACATAAAAAATAAAATATATATTGACATGGATTAAACAAAGATATAT<br>ATTATTTTATGTTGCGTAAACAAATTGGCAAAATAGAGATGGAAGATAAA<br>AATAAAAACCCTGGCAATAACCTACTCA
EPBBrown 107	*Borrelia bissettiae*	100%	TGAGTAGGTTATTGCCAGGGTTTTTATTTTTATATTTTAAATCTTGATTT<br>TATTTTATGTTTTTTAAATGTTTGTTAGTTTTTTTGAATGTGTTATTTAA<br>ATAACATAAAAAATAAAATATATATTGACATGGATTAAACAAAGATATAT<br>ATTATTTTATGTTGCGTAAACAAATTGGCAAAATAGAGATGGAAGATAAA<br>AATAAAAACCCTG
EPBBrown 110	*Borrelia burgdorferi* sensu stricto	100%	TGAGTAGGTTATTGCCAGGGTTTTTATTTTTATACTTTAAACTTTGATTT<br>TATTTTTATGTTTTTTAAATATTGGTGTTTTTGAATGTGTTGTTTAAATA<br>ACATAAAAAATAAAATATATATATTGACATGCATTAAACAAAGATATATA<br>TTATTTTATGTTGTATAAATAAATTGGCAAAATAGAGATGGAAGATAAAA<br>ATAAAAACCCTGG
EPBBrown 111	*Borrelia burgdorferi* sensu stricto	100%	TGAGTAGGTTATTGCCAGGGTTTTTATTTTTATACTTTAAACTTTGATTT<br>TATTTTTATGTTTTTTAAATATTGGTGTTTTTGAATGGGTTGTTTAAATA<br>ACATAAAAAATAAAATATATATATTGACATGCATTAAACAAAGATATATA<br>TTATTTTATGTTGTATAAATAAATTGGCAAAATAGAGATGGAAGATAAAA<br>ATAAAAACCCTGG
EPBBrown 128	*Borrelia burgdorferi* sensu stricto	100%	TGAGTAGGTTATTGCCAGGGTTTTTATTTTTATACTTTAAACTTTGATTT<br>TATTTTTATGTTTTTTAAATATTGGTGTTTTTGAATGTGTTGTTTAAATA<br>ACATAAAAAATAAAATATATATATTGACATGCATTAAACAAAGATATATA<br>TTATTTTATGTTGTATAAATAAATTGGCAAAATAGAGATGGAAGATAAAA<br>ATAAAAACCCTGG
EPBBrown 150	*Borrelia burgdorferi* sensu stricto	100%	TGAGTAGGTTATTGCCAGGGTTTTTATTTTTATACTTTAAACTTTGATTT<br>TATTTTTATGTTTTTTAAATATTGGTGTTTTTGAATGTGTTGTTTAAATA<br>ACATAAAAAATAAAATATATATATTGACATGCATTAAACAAAGATATATA<br>TTATTTTATGTTGTATAAATAAATTGGCAAAATAGAGATGGAAGATAAAA<br>ATAAAAACCC
EPBBrown 154	*Borrelia burgdorferi* sensu stricto	100%	TGAGTAGGTTATTGCCAGGGTTTTTATTTTTATACTTTAAACTTTGATTT<br>TATTTTTATGTTTTTTAAATATTGGTGTTTTTGAATGTGTTGTTTAAATA<br>ACATAAAAAATAAAATATATATTGACATGTATTAAACAAAGATATATATT<br>ATTTTATGTTGTATAAATAAATTGGCAAAATAGAGATGGAAGATAAAAAT<br>AAAAACCCTGGC
EPBBrown 162	*Borrelia burgdorferi* sensu stricto	100%	TGAGTAGGTTATTGCCAGGGTTTTTATTTTTATACTTTAAACTTTGATTT<br>TATTTTTATGTTTTTTAAATATTGGTGTTTTTGAATGGGTTGTTTAAATA<br>ACATAAAAAATAAAATATATATTGACATGCATTAAACAAAGATATATATT<br>ATTTTATGTTGTATAAATAAATTGGCAAAATAGAGATGGAAGATAAAAAT<br>AAAA
EPBBrown 169	*Borrelia burgdorferi* sensu stricto	100%	TGAGTAGGTTATTGCCAGGGTTTTTATTTTTATACTTTAAACTTTGATTT<br>TATTTTTATGTTTTTTAAATATTGGTGTTTTTGAATGTGTTGTTTAAATA<br>ACATAAAAAATAAAATATATATATTGACATGCATTAAACAAAGATATATA<br>TTATTTTATGTTGTATAAATAAATTGGCAAAATAGAGATGGAAGATAAAA<br>ATAAAAACC
EPBBrown 185	*Borrelia bissettiae*	100%	TGAGTAGGTTATTGCCAGGGTTTTTATTTTTATATTTTAAATCTTGATTT<br>TATTTTATGTTTTTTAAATGTTTGTTAGTTTTTTTGAATGTGTTATTTAA<br>ATAACATAAAAAATAAAATATATATTGACATGGATTAAACAAAGATATAT<br>ATTATTTTATGTTGCGTAAACAAATTGGCAAAATAGAGATGGAAGATAAA<br>AATAAAAA

EPBBrown 187	*Borrelia bissettiae*	100%	TGAGTAGGTTATTGCCAGGGTTTTTATTTTTATATTTTAAATCTTGATTT<br>TATTTTATGTTTTTTAAATGTTTGTTAGTTTTTTTGAATGTGTTATTTAA<br>ATAACATAAAAAAATAAAATATATATTGACATGGATTAAACAAAGATATAT<br>ATTATTTTATGTTGCGTAAACAAATTGGCAAAATAGAGATGGAAGATAAA<br>AATAAAAACCCTGGCAATAACCTACTCA
EPBBrown 189	*Borrelia bissettiae*	100%	TGAGTAGGTTATTGCCAGGGTTTTTATTTTTATATTTTAAATCTTGATTT<br>TATTTTATGTTTTTTAAATGTTTGTTAGTTTTTTTGAATGTGTTATTTAA<br>ATAACATAAAAAAATAAAATATATATTGACATGGATTAAACAAAGATATAT<br>ATTATTTTATGTTGCGTAAACAAATTGGCAAAATAGAGATGGAAGATAAA<br>AATAAAAA
EPBBrown 190	*Borrelia burgdorferi sensu lato*	N/A	N/A No clean sequence
EPBBrown 191	*Borrelia bissettiae*	100%	TGAGTAGGTTATTGCCAGGGTTTTTATTTTTATATTTTAAATCTTGATTT<br>TATTTTATGTTTTTTAAATGTTTGTTAGTTTTTTTGAATGTGTTATTTAA<br>ATAACATAAAAAAATAAAATATATATTGACATGGATTAAACAAAGATATAT<br>ATTATTTTATGTTGCGTAAACAAATTGGCAAAATAGAGATGGAAGATAAA<br>AATAAAAACCCTGGG
EPBBrown 197	*Borrelia burgdorferi sensu lato*	N/A	N/A No clean sequence
EPBBrown 213	*Borrelia bissettiae*	100%	TGAGTAGGTTATTGCCAGGGTTTTTATTTTTATATTTTAAATCTTGATTT<br>TATTTTATGTTTTTTAAATGTTAGTGTTTTTGAATATATTATTTAAATAA<br>CATAAAAAATAAAATATATATTGACATGGATTAAACAAAGATATATATTA<br>TTTTATGTTGCATAAACAAATTGGCAAAATAGAGATGGAAGATAAAAATA<br>AAAACCCTGG
EPBBrown 214	*Borrelia bissettiae*	100%	TGAGTAGGTTATTGCCAGGGTTTTTATTTTTATATTTTAAATCTTGATTT<br>TATTTTATGTTTTTTAAATGTTTGTTAGTTTTTTTGAATGTGTTATTTAA<br>ATAACATAAAAAAATAAAATATATATTGACATGGATTAAACAAAGATATAT<br>ATTATTTTATGTTGCGTAAACAAATTGGCAAAATAGAGATGGAAGATAAA<br>AATAAAAACCC
EPBBrown 216	*Borrelia bissettiae*	100%	TGAGTAGGTTATTGCCAGGGTTTTTATTTTTATATTTTAAATCTTGATTT<br>TATTTTATGTTTTTTAAATGTTTGTTAGTTTTTTTGAATGTGTTATTTAA<br>ATAACATAAAAAAATAAAATATATATTGACATGGATTAAACAAAGATATAT<br>ATTATTTTATGTTGCGTAAACAAATTGGCAAAATAGAGATGGAAGATAAA<br>AATAAAAA
EPBBrown 220	*Borrelia bissettiae*	100%	TGAGTAGGTTATTGCCAGGGTTTTTATTTTTATATTTTAAATCTTGATTT<br>TATTTTATGTTTTTTAAATGTTAGTGTTTTTGAATATATTATTTAAATAA<br>CATAAAAAATAAAATATATATTGACATGGATTAAACAAAGATATATATTA<br>TTTTATGTTGCATAAACAAATTGGCAAAATAGAGATGGAAGATAAAAATA<br>AAAACCCTGGGGCTAACC
EPBBrown 221	*Borrelia burgdorferi sensu lato*	N/A	N/A No clean sequence
EPBBrown 224	*Borrelia bissettiae*	100%	TGAGTAGGTTATTGCCAGGGTTTTTATTTTTATATTTTAAATCTTGATTT<br>TATTTTATGTTTTTTAAATGTTTGTTAGTTTTTTTGAATGTGTTATTTAA<br>ATAACATAAAAAAATAAAATATATATTGACATGGATTAAACAAAGATATAT<br>ATTATTTTATGTTGCGTAAACAAATTGGCAAAATAGAGATGGAAGATAAA<br>AATAAAAACCCTGGG
EPBBrown 229	*Borrelia bissettiae*	100%	TGAGTAGGTTATTGCCAGGGTTTTTATTTTTATATTTTAAATCTTGATTT<br>TATTTTATGTTTTTTAAATGTTAGTGTTTTTGAATATATTATTTAAATAA<br>CATAAAAAATAAAATATATATTGACATGGATTAAACAAAGATATATATTA<br>TTTTATGTTGCATAAACAAATTGGCAAAATAGAGATGGAAGATAAAAATA<br>AAAACCCTGGCAATAACCTACTCA

EPBBrown 231	*Borrelia burgdorferi* sensu stricto	100%	TGAGTAGGTTATTGCCAGGGTTTTTATTTTTATACTTTAAACTTTGATTT<br>TATTTTTATGTTTTTTAAATATTGGTGTTTTTGAATGTGTTGTTTAAATA<br>ACATAAAAAATAAAATATATATATTGACATGCATTAAACAAAGATATATA<br>TTATTTTATGTTGTATAAATAAATTGGCAAAATAGAGATGGAAGATAAAA<br>ATAAAAACCCTGGCG
EPBBrown 232	*Borrelia bissettiae*	100%	TGAGTAGGTTATTGCCAGGGTTTTTATTTTTATATTTTAAATCTTGATTT<br>TATTTTATGTTTTTTAAATGTTTGTTAGTTTTTTTGAATGTGTTATTTAA<br>ATAACATAAAAAATAAAATATATATTGACATGGATTAAACAAAGATATAT<br>ATTATTTTATGTTGCGTAAACAAATTGGCAAAATAGAGATGGAAGATAAA<br>AATAAAAAACCCTGG
EPBBrown 233	*Borrelia burgdorferi sensu lato*	N/A	N/A No clean sequence
EPBBrown 235	*Borrelia bissettiae*	100%	TGAGTAGGTTATTGCCAGGGTTTTTATTTTTATATTTTAAATCTTGATTT<br>TATTTTATGTTTTTTAAATGTTTGTTAGTTTTTTTGAATGTGTTATTTAA<br>ATAACATAAAAAATAAAATATATATTGACATGGATTAAACAAAGATATAT<br>ATTATTTTATGTTGCGTAAACAAATTGGCAAAATAGAGATGGAAGATAAA<br>AATAAAAAACCCTGG
EPBBrown 273	*Borrelia burgdorferi* sensu stricto	100%	TGAGTAGGTTATTGCCAGGGTTTTTATTTTTATACTTTAAACTTTGATTT<br>TATTTTTATGTTTTTTAAATATTGGTGTTTTTGAATGGGTTGTTTAAATA<br>ACATAAAAAATAAAATATATATATTGACATGCATTAAACAAAGATATATA<br>TTATTTTATGTTGTATAAATAAATTGGCAAAATAGAGATGGAAGATAAAA<br>ATAA
EPBBrown 287	*Borrelia burgdorferi* sensu stricto	99%	TGAGTAGGTTATTGCCAGGGTTTTTATTTTTATACTTTAAACTTTGATTT<br>TATTTTTATGTTTTTTAAATATTGGTGTTTTTGAATGTGTTGTTTAAATA<br>ACATAAAAAATAAAATATATAYATTGACATGCATTAAACAAAGATATATA<br>TTATTTTATGTTGTATAAATAAATTGGCAAAATAGAGATGGAAGATAAAA<br>ATAA
